# Cross-regulome profiling of RNA polymerases highlights the regulatory role of polymerase III on mRNA transcription by maintaining local chromatin architecture

**DOI:** 10.1186/s13059-022-02812-w

**Published:** 2022-11-28

**Authors:** Yongpeng Jiang, Jie Huang, Kai Tian, Xiao Yi, Haonan Zheng, Yi Zhu, Tiannan Guo, Xiong Ji

**Affiliations:** 1 Key Laboratory of Cell Proliferation and Differentiation of the Ministry of Education, School of Life Sciences, Peking-Tsinghua Center for Life Sciences, Peking University, Beijing, 100871 China; 2 Westlake Laboratory of Life Sciences and Biomedicine, Key Laboratory of Structural Biology of Zhejiang Province, School of Life Sciences, Westlake University, 18 Shilongshan Road, Hangzhou, 310024 Zhejiang Province China; 3 Institute of Basic Medical Sciences, Westlake Institute for Advanced Study, 18 Shilongshan Road, Hangzhou, 310024 Zhejiang Province China; 4 Westlake Omics (Hangzhou) Biotechnology Co., Ltd, Hangzhou, 310024 China

**Keywords:** RNA polymerases II and III, FACT complex, Local chromatin structure, Transcription rate

## Abstract

**Background:**

Mammalian cells have three types of RNA polymerases (Pols), Pol I, II, and III. However, the extent to which these polymerases are cross-regulated and the underlying mechanisms remain unclear.

**Results:**

We employ genome-wide profiling after acute depletion of Pol I, Pol II, or Pol III to assess cross-regulatory effects between these Pols. We find that these enzymes mainly affect the transcription of their own target genes, while certain genes are transcribed by the other polymerases. Importantly, the most active type of crosstalk is exemplified by the fact that Pol III depletion affects Pol II transcription. Pol II genes with transcription changes upon Pol III depletion are enriched in diverse cellular functions, and Pol III binding sites are found near their promoters. However, these Pol III binding sites do not correspond to transfer RNAs. Moreover, we demonstrate that Pol III regulates Pol II transcription and chromatin binding of the facilitates chromatin transcription (FACT) complex to alter local chromatin structures, which in turn affects the Pol II transcription rate.

**Conclusions:**

Our results support a model suggesting that RNA polymerases show cross-regulatory effects: Pol III affects local chromatin structures and the FACT-Pol II axis to regulate the Pol II transcription rate at certain gene loci. This study provides a new perspective for understanding the dysregulation of Pol III in various tissues affected by developmental diseases.

**Supplementary Information:**

The online version contains supplementary material available at 10.1186/s13059-022-02812-w.

## Background

RNA Pol I, Pol II, and Pol III were identified more than 50 years ago [1–4]. The conventional view indicates that Pol I transcribes ribosomal RNAs (rRNAs), Pol II transcribes messenger RNAs (mRNAs) and long noncoding RNAs (lncRNAs), and Pol III synthetizes transfer RNAs (tRNAs) and other small noncoding RNAs; moreover, these activities were initially identified in cells treated with different concentrations of a-amanitin [5–9]. Previously, low-resolution immunofluorescence labeling of RNA polymerases and nascent transcripts suggested that Pol I, Pol II, and Pol III were enriched at specific loci, which are also called transcription factories [10–13]. Mapping of the genomic localization of Pol II and Pol III revealed that they were closely associated [14–17]. However, according to our literature review, the genomic localization of Pol I, Pol II, and Pol III has never been simultaneously compared in the same cell type. Thus, Pol I-, Pol II-, and Pol III-specific genes and cross-regulated genes have not been systematically characterized.

Recent studies have shown cross-regulation among different Pols at specific genes. For example, Pol II-mediated formation of R-loops prevents Pol I transcription of large intergenic spacer (IGS) regions in the nucleolus [18]. Transcriptional interference has been identified between Pol II and downstream Pol III-transcribed tRNAs [19], and cross-regulation by shared transcriptional regulators or noncoding RNAs has been suggested [20–25]. Pol III-occupied transposon elements have been proposed to be enhancers of neighboring protein-coding genes [26, 27]. Specifically, the short interspersed element (SINE) adjacent to the *Fos* gene transcribes enhancer RNA to regulate *Fos* expression in neurons [20]. Unfortunately, the mechanisms of transcriptional interference and noncoding RNAs have been identified primarily in studies of specific genes. It is unclear whether these mechanisms are generalizable. In particular, the cross-regulatory roles played by Pol III are still unclear.

Pol III plays a critical role in various biological processes. For example, a reduction in Pol III transcription leads to lifespan extension in yeast and flies [28], and Pol III is required for hypertrophic growth and transformation in different types of cancers [29–33]. Mutations in Pol III subunits lead to developmental defects in different tissues; for example, RPC1 and RPC2 mutations are associated with hypomyelinating leukodystrophy [34], RPC8 mutations cause primary ovarian insufficiency [35], and mutations in RPAC1 and RPAC2 have been identified in craniofacial development disorders [36, 37]. However, an explanation of how constitutive transcriptional activities of Pol III lead to its diverse functions in different tissues is lacking.

We previously achieved rapid depletion of the largest individual subunits of Pol I (RPA1), Pol II (RPB1), and Pol III (RPC1) using an auxin-inducible degron system and showed that these RNA polymerases played roles in mediating local, small-scale 3D chromatin structural changes [38]. Considering advantage of these degron systems, we performed precision nuclear run-on sequencing (PRO-Seq), assay for transposase-accessible chromatin sequencing (ATAC-Seq), and chromatin immunoprecipitation sequencing (ChIP-Seq) experiments for the other two Pols after individual depletion of each Pol. We further performed chromatin-associated RNA sequencing (ChAR-Seq) and Pol II ChIP followed by mass spectrometry (ChIP-MS) after Pol III depletion and Pol II ChIP-Seq analyses after depletion of a small Pol III subunit (RPAC1). By performing integrated analyses of these large-scale datasets, we found that Pol III is required for nucleosome destabilization and FACT recruitment and regulates the Pol II transcription rate of nearby mRNA genes. Our results indicate that Pol III plays a crucial role in maintaining an active chromatin architecture and facilitating the transcription of nearby mRNA genes.

## Results

### Disruption of Pol I, Pol II, or Pol III transcription in mouse embryonic stem cells (mESCs)

**Fig. 1:**
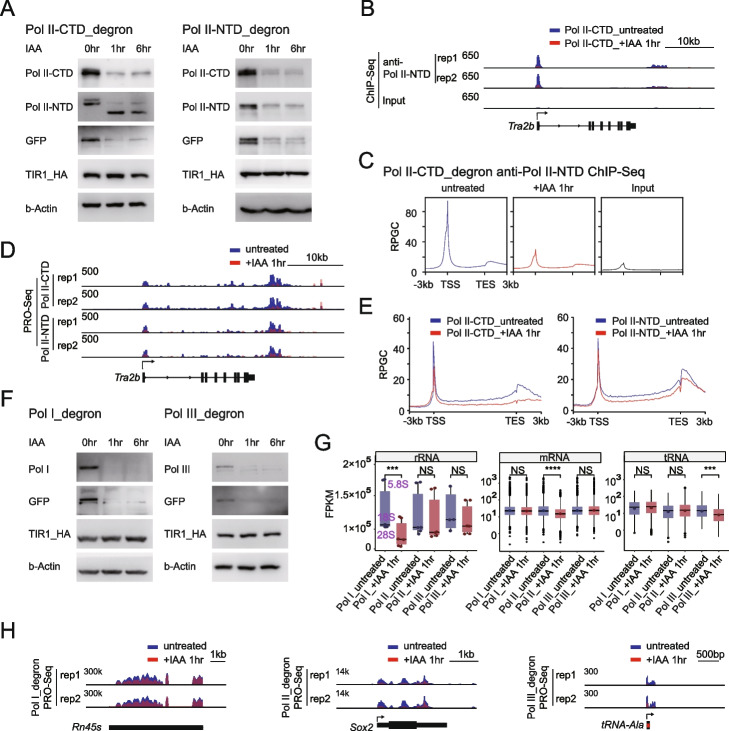
Rapid disruption of Pol I, Pol II, and Pol III transcription in mESCs. **A** Western blot analyses of Pol II (RPB1) protein levels at different time points after IAA treatment in C-terminal domain (CTD, left) and N-terminal domain (NTD, right) degron mESCs. GFP was fused to the degron tag and used to confirm degradation. HA-tagged TIR1 (TIR1-HA) level were also examined to indicate efficient induction of TIR1. b-Actin served as the loading control. **B** Genome browser ChIP-Seq track at the 22,193,658–22,321,486 region on chromosome 16 in Pol II_CTD_degron cells under untreated (upper) and after 1 h of IAA treatment conditions (middle). ChIP-Seq was performed with an antibody recognizing the Pol II (RPB1) N-terminal (anti-Pol II-NTD). The input is shown in the bottom panel, all tracks are flipped horizontally, and the *y*-axis shows the normalized read density in reads per genome coverage (RPGC). **C** Average metagene profiles of Pol II occupancy on gene bodies and the adjacent regions 3 kb upstream and downstream in Pol II_CTD_degron cells at active mRNA genes under untreated (left) and after 1 h of IAA treatment conditions (middle) with an antibody recognizing the Pol II (RPB1) N-terminal (anti-Pol II-NTD). The input is shown in the right panel. **D** Genome browser PRO-Seq track at the same region described in Fig. 1B in Pol II_CTD_degron and Pol II_NTD_degron cells under untreated and after 1 h of IAA treatment conditions. The *y*-axis shows the normalized read density in reads per kilobase per million mapped reads (RPKM). Only sense strand signals are presented, and all tracks are flipped horizontally. **E** Average metagene profiles of spike-in-normalized PRO-Seq signals at active mRNA genes in Pol II_CTD_degron (left) and Pol II_NTD_degron (right) cells under untreated and after 1 h of IAA treatment conditions. **F** Western blot analysis of Pol I (RPA1) and Pol III (RPC1) protein levels at different time points after IAA treatment. Immunoblotting was performed with antibodies recognizing the N-terminal domains, as shown in Fig. 1A. **G** Normalized PRO-Seq read counts were summed and compared at rRNA (left, *N* = 3), active mRNAs (middle, *N* = 8845), and tRNA loci (right, *N* = 435) in Pol I degron, Pol II CTD_degron, and Pol III degron cells under untreated and after 1 h of IAA treatment conditions. The rRNA density was calculated as the sum of the 5.8S, 18S, and 28S rRNA densities. The boxplots show the range of the values, with the median indicated by a line. The whiskers on the boxplots show the lowest data value within IQR=1.5 of the lower quartile and the highest data value within IQR=1.5 of the upper quartile. The *p* value was calculated using the Mann–Whitney test. **H** Genome browser PRO-Seq track at the 39,840,997–39,850,829 region on chromosome 17, 34,648,531–34,653,537 region on chromosome 3 and 21,240,180–21,243,656 region on chromosome 13 in Pol I_degron, Pol II_CTD_degron, and Pol III_degron cells under untreated and after 1 h of IAA treatment conditions. The *y*-axis shows the normalized read density in reads per kilobase per million mapped reads (RPKM). Only sense strand signals are presented

A recent study indicated that insertion of a degron tag into the carboxy-terminal domain (CTD) of RPB1 (Pol II) leads only to degradation of the RPB1 CTD but not full-length RPB1 [19, 39]. We confirmed this finding and showed that CTD depletion (Fig. 1A) dramatically decreased Pol II transcription by performing both RPB1 ChIP-Seq with an antibody recognizing the amino (N)-terminus (Fig. 1B, C) and PRO-Seq (Fig. 1D, E) at the representative gene and genome-wide levels. In addition, we inserted a degron tag into the N-terminus of RPB1, which induced the degradation of full-length RPB1 (Fig. 1A) and inhibition of Pol II transcription (Fig. 1D,E). Since there was no difference in the degree of transcriptional repression with either RPB1 CTD or amino-terminal domain (NTD) depleted, we used the RPB1 CTD degron cell line in the rest of our study.

PRO-Seq was performed to ensure the efficient disruption of Pol I, Pol II, and Pol III transcription with our degron system. Western blotting with antibodies recognizing the NTD of the largest Pol I and Pol III subunits revealed the successful depletion of full-length RPA1 (Pol I) and RPC1 (Pol III) with our degron system (Fig. 1F). Then, PRO-Seq was performed after Pol I, Pol II, or Pol III depletion for 1 h when obvious depletion of a given Pol was achieved. Pol I depletion led to a minimal effect on the production of mRNAs or tRNAs but significantly suppressed the transcription of rRNA genes. Similarly, preferential decreases in mRNA and tRNA transcripts were observed after depletion of Pol II and Pol III, respectively (Fig. 1G). The PRO-Seq signals at specific gene loci further confirmed their functional repression upon polymerase depletion (Fig. 1H). These results indicated that we rapidly disrupted Pol I, Pol II, and Pol III transcription with the degron system in mESCs.

### Pol I, Pol II, and Pol III mostly occupy specific genomic regions, but close associations exist at specific loci

We generated Pol I, Pol II, and Pol III ChIP-Seq datasets to investigate the relationship of their genomic occupancy. As expected, Pol I, Pol II, and Pol III ChIP-seq signals were differentially enriched at loci producing known RNAs such as rRNAs, mRNAs, and tRNAs (Additional file 1: Fig. S1A), and the chromatin binding of different Pols significantly decreased at their peaks after their depletion, as we showed previously [38]. To rule out the possibility that mapping artifacts might have caused ChIP-Seq peak overlaps of all three Pols, we performed a quality examination of the aligned reads [38]. Following the quality control (QC) measures of the ENCODE consortia (Additional file 1: Fig. S1B-D), we obtained reliable binding peaks for the three Pols. A heatmap cluster analysis of the ChIP-Seq data indicated that Pol I, Pol II, and Pol III bound mostly to specific genomic regions and that only a few sites were co-occupied. Quantification analysis showed that the average Pol I ChIP-Seq signal was 1.71- and 2.77-fold more intense than the Pol II and Pol III signals in cluster 1, respectively; the average Pol II ChIP-Seq signal was 4.73- and 5.85-fold more intense than the Pol I and Pol III signals in cluster 2, respectively; and the average Pol III ChIP-Seq signal was 3.5- and 2.91-fold more intense than the Pol I and Pol II signals in cluster 3, respectively. Specifically, Pol II and Pol III co-occupied many sites (*n*=695) in the genome (Additional file 1: Fig. S1E), consistent with previous reports [14–17]. In total, 191 sites of Pol I and Pol II overlapped, 45 sites of Pol I and Pol III overlapped, and 153 sites of Pol I, Pol II, and Pol III overlapped (Additional file 1: Fig. S1E); the genes at sites where all three Pols overlapped were enriched in transcriptional regulation, cell cycle, and mRNA processing functions. Detailed analysis of the genomic distribution of each cluster revealed that Pol III-specific targets were preferentially associated with short genes, such as tRNAs and retrotransposon elements (including SINEs, long interspersed elements (LINEs), and long terminal repeats (LTRs)), while the remaining clusters exhibited preferential occupancy in mRNA regions (Additional file 1: Fig. S1F). In addition, genomic correlation analyses indicated that Pol I, Pol II, and Pol III peaks were positively correlated with active histone modification markers (Additional file 1: Fig. S1G, Additional file 2: Table S1). Collectively, these results suggest that the majority of Pol targets are specific and that only a small fraction are closely associated. The close chromatin binding of different Pols at specific loci may create opportunities for biologically meaningful cross-regulatory effects, as reported previously [23, 40–43].

To compare the proteomic landscape of chromatin in the context of different Pols, we carried out ChIP coupled to quantitative MS. The results showed that the Pol I-, Pol II-, and Pol III-specific subunits were preferentially enriched in their corresponding ChIP-MS preparations; the subunits shared by Pol I and Pol III (RPAC1 and RPAC2) and the subunits shared by all three Pols (RPB5, RPB6, RPB8, RPB10, and RPB12) were also preferentially detected, as expected (Fig. S2A-B, Additional file 3: Table S2) [44]. Transcriptional processes are known to be extensively regulated by trans-acting factors and cis-regulatory elements [45–49], and ChIP-MS detected Pol II transcriptional regulators involved in initiation, elongation, and termination processes. Interestingly, many of these regulators were also detected in the Pol I and Pol III ChIP-MS preparations (Fig. S2A-B, Additional file 3: Table S2). However, some contamination during the formaldehyde crosslinking process cannot be ruled out. It is also reminiscent of the previous observation that Pols could be found in close association on the chromatin [14–17], where some transcriptional regulators may coordinate the transcription by different Pols [23, 40–43].

### Orthogonal experimental analyses revealed the predominant role played by Pol III in the regulation of Pol II transcription

**Fig. 2:**
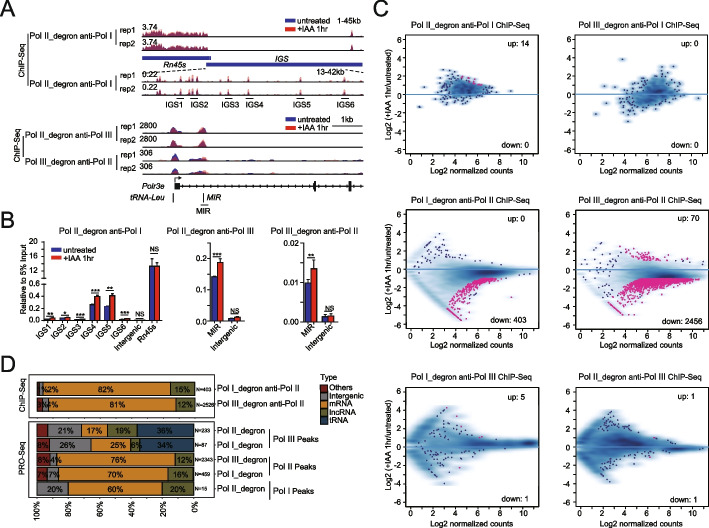
Orthogonal experimental analyses revealed the predominant role of Pol III in the regulation of Pol II transcription. **A** Genome browser track of Pol I ChIP-Seq signals at rRNA cDNA units in Pol II_degron cells under untreated conditions and after 1 h of IAA treatment to visualize the IGS regions (see “Methods” for details, top). Genome browser track of Pol II ChIP-Seq signals in Pol III_degron cells or Pol III ChIP-Seq signals in Pol II_degron cells in the 120,914,905–120,924,630 region on chromosome 7 under untreated conditions and after 1 h of IAA treatment (bottom). The *y*-axis shows the normalized read density in reads per genome coverage (RPGC). Note that the intergenic spacer (IGS) region is magnified at the bottom to show the details. Two biological replicates are shown. **B** Bar graphs showing the relative ChIP enrichment normalized to input (5%) at the loci indicated in Fig. 2A (Additional file 12: Table S11). Each sample was analyzed with two technical replicates per biological replicate and two biological replicates in total. Statistical significance was evaluated by Student’s *t* test (***: <0.001, **: <0.01, *: <0.05, NS: not significant). **C** MA plots showing differential enrichment of Pol I ChIP-Seq signals around Pol I-bound peaks in Pol II_degron (left) and Pol III_degron (right) cells under untreated and after 1 h of IAA treatment conditions (upper). Each dot represents one peak. Red indicates a significant change that meets both criteria of a false discovery rate (FDR) < 0.05 and fold change > 2. Similarly, Pol II ChIP-Seq performed in Pol I_degron (left) and Pol III_degron (right) cells (middle) and Pol III ChIP-Seq performed in Pol I_degron (left) and Pol II_degron (right) cells (bottom) are presented. **D** Horizontally stacked bar charts showing the genomic distribution of differential peaks identified by Pol II ChIP-Seq and evaluated by factor perturbation analysis (upper panel, data from Fig. 2C). These results were further confirmed by conducting a similar analysis at the transcriptional level using PRO-Seq data from factor perturbation experiments (bottom)

The observed colocalization of Pols and the identification of common transcriptional regulators, however, do not necessarily indicate cross-regulation among different Pols. Therefore, we performed orthogonal experimental analyses of ChIP-Seq data of the Pols after Pol I, Pol II, or Pol III depletion for 1 h to examine the immediate response, which mitigate concerns that secondary effects affected the measurements. Our sequencing data confirmed the known cross-regulatory relationships among different Pols. For example, Pol II inhibition was recently reported to increase Pol I transcription at IGS regions in rRNA loci [18], and in our study, Pol I ChIP-Seq signals showed an increase in Pol I binding at IGS loci after Pol II depletion (Fig. 2A). The MIR gene, located at the Polr3e locus, is transcribed by Pol III, and transcriptional interference of Pol II and Pol III at the MIR and Polr3e loci has been found [19, 50]. Consistent with this report, our study showed that Pol II depletion increased Pol III chromatin binding at the MIR locus. Interestingly, Pol III depletion increased the Pol II ChIP signals at the MIR locus (Fig. 2A), possibly due to the aforementioned interference of Pol III and Pol II transcription on this gene. ChIP-Seq showed changes at targeted sites, and these findings were further validated by ChIP–qPCR (Fig. 2B). Together, these results suggest that our PRO-Seq and ChIP-Seq datasets are reliable and biologically relevant (Additional file 4: Table S3).

To systemically identify the targets cross-regulated by different Pols, we performed differential analyses with the cross-regulome ChIP-Seq and PRO-Seq datasets. The differential analyses were initially performed with the cross-regulome ChIP-Seq signals at the peaks of corresponding Pols in wild-type cells. An analysis of the MA plots revealed that the Pol II ChIP-Seq signals changed the most dramatically after Pol III depletion (Additional file 5: Table S4). The next greatest change was observed in the Pol II ChIP-Seq signals after Pol I depletion (Fig. 2C). We then performed differential analyses of PRO-Seq signal changes at different Pol-bound peaks. The results showed that the two most affected categories were the same as those identified with the ChIP-Seq dataset (Fig. 2D). PRO-Seq and Pol II ChIP-Seq after Pol III depletion showed that the significantly altered sites overlapped more with Pol III ChIP-Seq peaks than with other Pols peaks (Additional file 5: Table S4 and Additional file 6: Table S5). Hence, for the rest of this study, we mainly focused on the roles of Pol III in Pol II transcriptional regulation.

### Pol III depletion perturbs Pol II transcription in the gene body

**Fig. 3:**
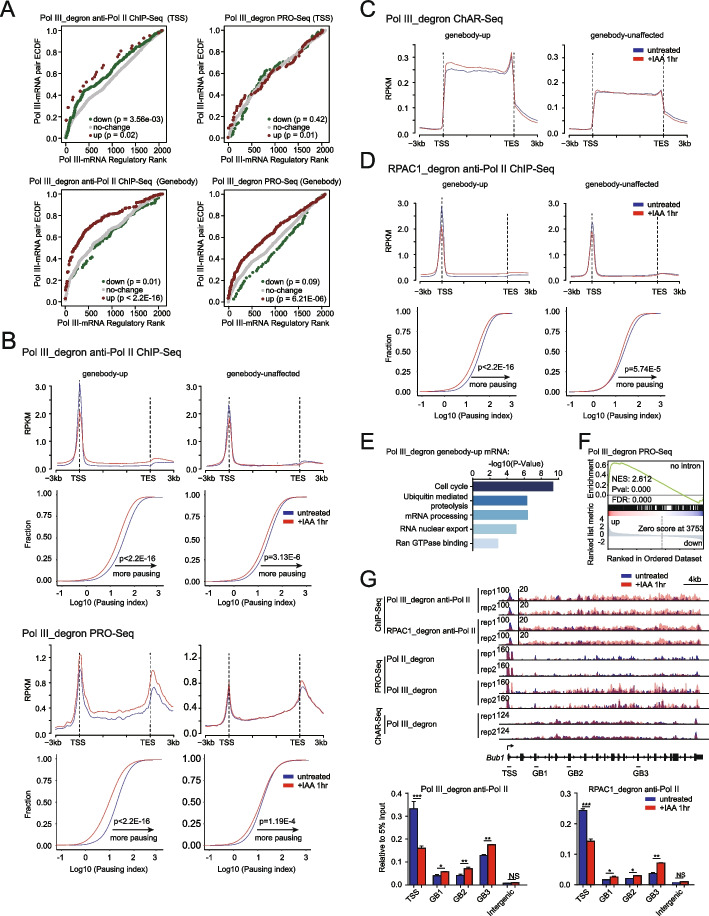
Pol III depletion perturbs the transcription of Pol II at gene bodies. **A** Modified beta analysis results depicting the activating and repressive functions of Pol III binding events. The red, green, and gray dots represent cumulative fractions of mRNA genes that were upregulated, downregulated, or unchanged by Pol III depletion based on the Pol II ChIP-Seq (left) or PRO-Seq (right) results. Genes were ranked from high to low according to the corresponding Pol III peaks (see “Methods”). *P* values were calculated by two-sided Kolmogorov–Smirnov tests to determine whether the up- or downregulated groups differed significantly from the control group of transcriptionally unchanged genes. Integrated DNA-binding and target expression analysis from PRO-Seq or Pol II ChIP-Seq data after Pol III perturbation revealed that Pol III loss of function was mainly responsible for the significant upregulation of Pol III-associated genes within gene bodies but had little correlation with downregulated genes. **B** Top panel: Average metagene profiles of Pol II ChIP-Seq or PRO-Seq signals (normalized reads per million) on gene bodies and the adjacent regions 3 kb upstream and downstream in Pol III_degron cells subjected to 1 h IAA treatment versus untreated conditions: genebody-up (left, *N*=773) and genebody-unaffected mRNA (right, *N*=3290). Bottom panel: Empirical cumulative density function (ECDF) plots of the Pol II pausing index between Pol III_degron cells under 1 h IAA treatment and untreated conditions for each gene set as described above. Statistical significance was evaluated by the two-sided Wilcoxon rank-sum test. **C** Average metagene profiles of spike-in-normalized chromatin-associated RNA-Seq (ChAR-Seq) signals on gene bodies and the adjacent regions 3 kb upstream and downstream in Pol III_degron cells with 1 h of IAA treatment versus untreated mESCs for the same gene sets referenced in Fig. 3B. Remarkably, the overall increase in the gene body signals of genebody-up genes was highly consistent with the similar trend observed in the PRO-Seq data. **D** Analysis described in Fig. 3B for RPAC1 degron in mESCs. **E** Gene ontology analysis for the identified genes with genebody-up Pol II genes after depletion of Pol III (*n* = 773). **F** Gene set enrichment analysis (GSEA) revealed the enrichment of intronless genes by PRO-Seq analysis of gene expression changes in Pol III_degron cells. The normalized enrichment score (NES) and nominal *p* value were calculated using the GSEA package with 1000 permutations. **G** Genome browser track at the 127,798,200–127,833,859 region on chromosome 2 for Pol II ChIP-Seq signals in Pol III_degron and RPAC1_degron cells that were untreated or treated with IAA for 1 h. The genome browser track at the same region for PRO-Seq and chromatin-associated RNA-Seq signals in PoI III_degron cells that were untreated or treated with IAA for 1 h are also shown. The *y*-axis in the ChIP-Seq plot shows the normalized read density in reads per genome coverage (RPGC), and the *y*-axes in the PRO-Seq and ChAR-Seq plots show the normalized read density in reads per kilobase per million mapped reads (RPKM). Note that the Bub1 gene body (GB) region is magnified with a set scale to show the details, and all tracks are flipped horizontally. Bottom panel: Bar graphs showing relative ChIP enrichment normalized to input (5%) at the locus indicated above (Additional file 12: Table S11). Each sample was analyzed with two technical replicates per biological replicate and two biological replicates in total. Statistical significance was evaluated by Student’s *t* test (***: <0.001, **: <0.01, *: <0.05, NS: not significant). Only sense strand signals of PRO-Seq are presented

We assessed the changes in the ChIP-Seq and PRO-Seq signals at promoters and throughout gene bodies to gain mechanistic insights into the effects of Pol III on Pol II transcription. To examine the direct effects of Pol III on Pol II-transcribed mRNA genes, we established two interaction models: a 3D spatial proximity model based on histone H3 acetylated on lysine 27 (H3K27ac) HiChIP loops obtained from previous study and a model based on the linear regulation of the nearest mRNA genes. The Pol III peaks were assigned to Pol III-interacting mRNA genes with interactions identified by H3K27ac HiChIP data or to the nearest active mRNA genes (without interactions identified through H3K27ac HiChIP data) to identify Pol III-mRNA gene pairs. The correlation between changes in ChIP-Seq or PRO-Seq signals at these mRNA genes and the paired Pol III ChIP-Seq signals were plotted on a receiver operator characteristic (ROC) curve. The results showed that Pol III depletion caused a significant decrease in Pol II ChIP-Seq signals at transcription start sites (TSSs) and an increase in these signals in gene body regions, while the PRO-Seq signals were increased in gene body regions (Fig. 3A), suggesting that Pol III depletion perturbs Pol II transcription on the gene body of mRNA genes; these findings were consistent in both the Pol II ChIP-Seq analysis and the PRO-Seq analysis.

Genes with increased Pol II ChIP-Seq and PRO-Seq signals in the gene body upon Pol III depletion were designated genebody-up genes, and genes with unchanged Pol II ChIP-Seq and PRO-Seq signals in the gene body upon Pol III depletion were designated genebody-unaffected genes (Additional file 6: Table S5). The metagene analyses showed that Pol II ChIP signals decreased at TSSs and increased in gene body regions, that PRO-Seq signals increased both at TSSs and in gene body regions, that the pausing index of both the ChIP-Seq and PRO-Seq signals for genebody-up genes was significantly decreased, and that genebody-unaffected genes exhibited with fewer changes in PRO-Seq signals and slight decreases in Pol II ChIP-Seq signals at TSSs (Fig. 3B). These results suggest that depletion of Pol III leads to a widespread decrease of promoter-proximal pausing and increased Pol II signals at gene bodies for a subset of genes, as we described. PRO-seq signals at the promoter-proximal regions increased, which may be due to insufficient fragmentation during library preparation of PRO-seq causing promoter-proximal region-derived RNA to be captured with gene body-derived RNA. Furthermore, ChAR-Seq after Pol III depletion confirmed that the signals increased in gene body regions of genebody-up genes, while those of the genebody-unaffected genes were not obviously changed (Fig. 3C). Consistent with these results, depletion of the Pol III small subunit RPAC1 also decreased Pol II ChIP signals at TSSs, increased these signals in gene body regions, decreased the pausing index of the genebody-up genes, and showed fewer effects on Pol II ChIP signals in gene body regions and on the pausing index of the genebody-unaffected genes (Fig. 3D, Additional file 1: Fig. S2C). Together, our results demonstrated that Pol III is required for the proper transcription of a subset of Pol II-transcribed genes in mESCs.

The molecular features of Pol II-transcribed genes with Pol II signals in gene body regions affected by Pol III were characterized. Gene Ontology (GO) analysis indicated that Pol II genebody-up genes were enriched in the cell cycle, ubiquitin-mediated proteolysis, mRNA processing, and Ran GTPase binding functions (Fig. 3E). Additionally, gene set enrichment analysis (GSEA) indicated that a set of genes with upregulated PRO-Seq signals after Pol III depletion was enriched with intronless genes (Fig. 3F). Studies of these cross-regulated genes could expand the understanding of the diverse functions of Pol III in different biological systems in the future. For example, the serine/threonine protein kinase Bub1 is a key regulator of the cell cycle [51, 52]. Pol III depletion slightly increased the Pol II ChIP signal, nascent RNA, and chromatin-associated RNA levels at its gene body. The ChIP-Seq changes for Bub1 were independently validated with Pol II ChIP–qPCR after both Pol III and RPAC1 depletion (Fig. 3G). Moreover, cell cycle genes regulated by Pol III are consistent with the previous observation that Pol III assembly is associated with cell cycle progression [53].

### Pol III depletion affects the nucleosome occupancy of nearby mRNA genes

**Fig. 4:**
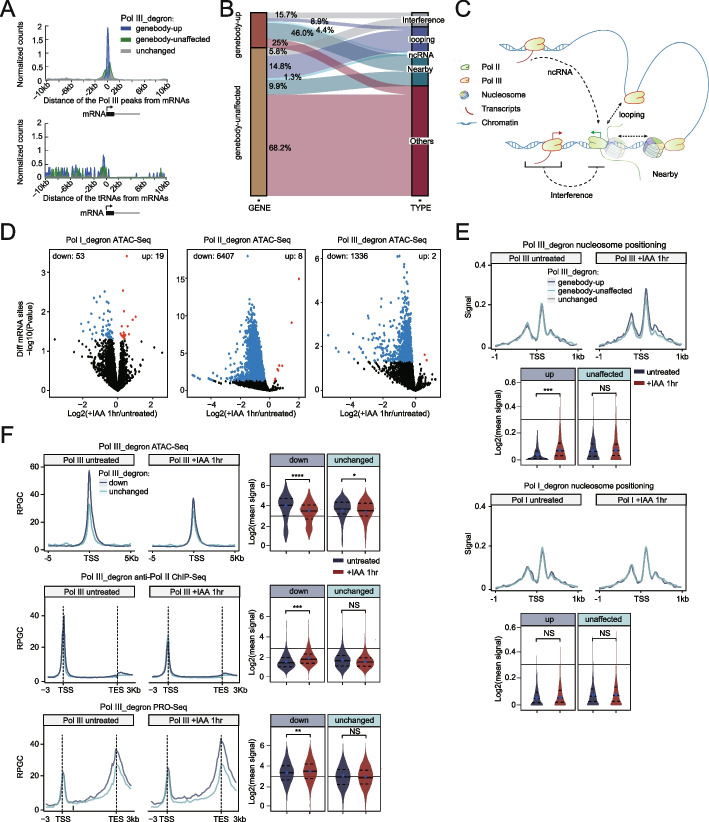
Pol III depletion affects the nucleosome occupancy of nearby mRNA genes. **A** Genebody-up mRNAs (blue), genebody-unaffected mRNAs (green), and unchanged mRNAs upon Pol III depletion (control, gray, unchanged genes) were defined by selecting the genes that were not at all affected by Pol III depletion; for details, see “Methods”) were clustered based on their distance from the nearest Pol III-bound peaks (upper) and expressed tRNAs (bottom) in 50 bp bins. **B** Sankey plot depicting relationships between Pol III and Pol II for the genebody-up genes and genebody-unaffected genes after Pol III depletion and four hypotheses in the subgroup, namely, transcription interference (interference), 3D interactions (looping), noncoding RNA (ncRNA), and nearby models. For detailed definitions, see Fig. S2E. **C** Working model for the cross-regulatory relationship between Pol III and Pol II by transcriptional interference, noncoding RNAs, 3D chromatin looping, and they occupy nearby but could not be explained the mechanisms listed above. **D** Volcano plots showing differentially accessible regions within active mRNA promoters (*N*=8845, ±1 kb centered on the TSS) identified by ATAC-seq upon depletion of Pol I, Pol II, and Pol III. The red dots represent a significant increase in chromatin accessibility, whereas the blue dots represent a significant decrease (adjusted *P* < 0.05). **E** ATAC-seq metaplots of mononucleosomes over the promoter regions of genebody-up mRNAs (*N* = 773, blue), genebody-unaffected mRNAs (*N* = 3290, green), and unchanged mRNAs (control, gray, for the definition, see “Methods”) upon Pol III depletion in Pol III_degron and Pol I_degron cells that were untreated or treated with IAA for 1 h (upper panel). Violin plots measuring the changes in promoter-proximal ATAC-seq mononucleosome signals over genebody-up or genebody-unaffected mRNA genes (bottom panel). Each violin plot shows the range of values, with the median indicated by a blue dot. The *p* value was calculated using the Mann–Whitney test. **F** Top panel: Composite metagene analysis of ATAC-Seq (upper) around the promoters, Pol II ChIP-Seq (middle), and PRO-Seq signals (bottom) around the gene bodies of mRNA genes in Pol III_degron cells with decreased and unchanged chromatin accessibility upon Pol III depletion, as shown in Fig. 4D. Promoters were defined as the regions within ± 2 kb of the transcription start site. For each metagene plot, the average profile (*Y*-axis) is displayed in normalized reads per million (RPM). Bottom panel: Violin plots (right) showing the quantification of ATAC-Seq, Pol II ChIP-Seq, and PRO-Seq signal changes for each group of gene sets shown in the top panel. The *p* value was calculated using the Mann–Whitney test

We next explored how Pol III regulates mRNA gene expression. Notably, the relationship between the genomic positions of Pol III binding sites and their regulation of mRNA gene transcription is unclear. The Pol III ChIP-Seq peaks and tRNA signals at the TSS of Pol II genebody-up and Pol II genebody-unaffected mRNA genes were plotted. The results showed that the mRNA genes affected by Pol III exhibited nearby Pol III peaks, but the tRNA annotations appeared to not be highly enriched (Fig. 4A), implying that Pol III may contribute to the regulation of Pol II transcription of RNAs other than tRNAs. The PRO-Seq and EU-Seq signals at all Pol III peaks and the specific regions consistently showed a noticeable decrease after 1 h of depletion of Pol III (Additional file 1: Fig. S2D), implying the reliability of the identified Pol III peaks. We envisioned that in-depth analyses of Pol III-affected Pol II genes might lead to greater insights into the mechanistic details of the Pol III effects on Pol II transcription. To determine the number of Pol II genes that may be regulated by Pol III through transcriptional interference, we investigated the relative positions of Pol II and Pol III chromatin binding. There are two possibilities: (a) Pol II and Pol III binding regions overlap, enabling the Pols to interfere directly with the transcription of each other; (b) Pol II and Pol III binding sites do not overlap but are very close to one another, which may affect transcription at the corresponding sites via sense or antisense transcriptional readthrough. The SINE elements encode Pol III-transcribed noncoding RNAs, which were proposed to regulate Pol II transcription through chromatin looping [20, 27]. Therefore, we analyzed the relationships of Pol III peaks and Pol II peaks with noncoding RNAs or chromatin looping (Additional file 1: Fig. S2E-F, the details for the sequential subgroup analysis are provided in the “Methods” section). We then annotated the Pol III binding sites associated with the regulation of Pol II activity and identified 121 (15.7%), 68 (8.9%), and 34 (4.4%) genebody-up genes (*N*=773). We speculated that these genes are regulated by Pol III, which may be explained by transcriptional interference, chromatin looping, and ncRNA models, respectively. Interestingly, we identified 356 (46%) genebody-up genes with nearby Pol III peaks (Fig. 4B,C), which could not be directly explained by the mechanisms of transcriptional interference, chromatin looping, and noncoding RNA involvement.

Our previous results and those of others have shown that Pols contribute to local 3D chromatin organization [38, 54], and we envisioned that these Pols might also play roles in local 1D chromatin architecture, i.e., nucleosome positioning. Therefore, we carried out ATAC-Seq after depleting each Pol for 1 h, as this assay can provide information about both nucleosome positioning and chromatin accessibility. Differential analyses of the ATAC-Seq signals at mRNA promoters were performed. The volcano plot shows that Pol II and Pol III loss led to a marked decrease in chromatin accessibility, while Pol I loss appeared to induce much less dramatic effects (Fig. 4D). These results suggest that Pol II and Pol III broadly affect nucleosome structures in the genome but that Pol I has less impact, possibly because of its restricted location within the nucleolus.

We analyzed our ATAC-Seq to obtain similar information by following the pipelines described in previous studies [55, 56]. The nucleosome positioning at genebody-up genes was increased more than that for genebody-unaffected genes after Pol III depletion. The increase in nucleosome occupancy might reduce the rate of Pol II transcription, thus increasing the densities of Pol II and PRO-Seq signals. As a control, nucleosome positioning of these two groups of genes was assessed after Pol I depletion, and no apparent changes were observed (Fig. 4E). The increase in nucleosome positioning was correlated with a decrease in chromatin accessibility (Additional file 1: Fig. S2G, Additional file 7: Table S6). On the other hand, meta-analyses of the genes with significantly decreased ATAC-Seq signals revealed an increase in Pol II signals in gene body regions in both the ChIP-Seq and PRO-Seq datasets after Pol III depletion (Fig. 4F). Additionally, Pol III depletion did not affect Pol II (RPB1) interactions with other Pol II subunits, suggesting that the composition of the Pol II complex remained stable after Pol III depletion (Additional file 1: Fig. S2H). These results suggest that Pol III destabilizes local nucleosome occupancy, providing molecular insights into Pol III-mediated transcriptional regulation of nearby mRNA genes.

### Local nucleosome positioning changes helps to explain the effects of Pol II on Pol III transcription

**Fig. 5:**
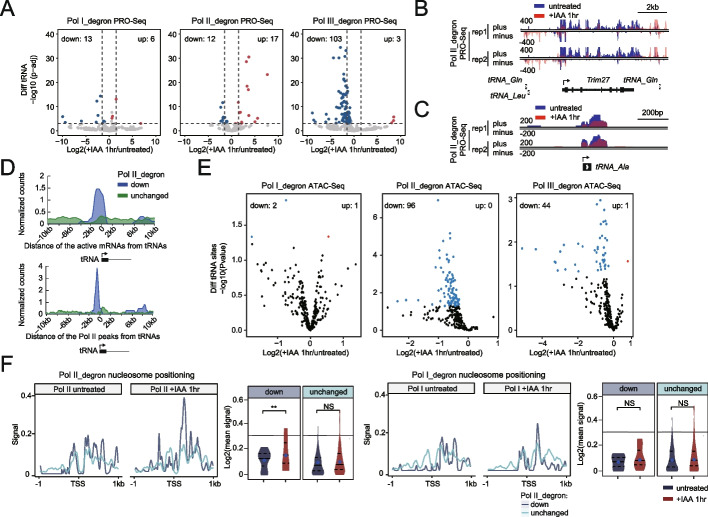
Local nucleosome positioning changes helps to explain the effects of Pol II on tRNA transcription. **A** Volcano plots showing the PRO-Seq signal changes over tRNA genes (*N* = 435) upon depletion of Pol I, Pol II, or Pol III. Statistically significant changes were determined from two biological replicates by the threshold criteria of an adjusted *p* value < 0.05 and absolute log2(fold change) >1 using DESeq2, with red representing upregulated genes and blue representing downregulated genes. **B** PRO-Seq in Pol II_degron cells under untreated conditions and after 1 h of IAA treatment. The Genome Browser track at the 21,166,370–21,202,236 region on chromosome 13 is shown with a bidirectional transcription signal. The *y*-axis shows the normalized read density in reads per kilobase per million mapped reads (RPKM). **C** The same data as described in Fig. 5B are shown for the 21,239,788–21,242,246 region on chromosome 13. **D** Under the same representations described in Fig. 4A, downregulated (*N* = 12, blue) and unchanged tRNA genes (*N* = 244, green) upon Pol II depletion in PRO-Seq were clustered based on their distance from the nearest expressed mRNAs and Pol II-bound peaks in 50-bp bins. **E** Under the same conditions described in Fig. 4D, differentially accessible sites around tRNA loci (*N* = 435, ±1 kb region centered on the tRNA genes) upon depletion of Pol I, Pol II, and Pol III are shown. **F** The ATAC-seq metaplots and violin plots shown are the same as those described in Fig. 4E but are based on the downregulated or unchanged tRNA genes upon Pol II depletion identified by PRO-Seq. The *p* value was calculated using the Mann–Whitney test

We also assessed tRNA expression by analyzing PRO-Seq data after loss of Pol I, Pol II, or Pol III. As expected, Pol III depletion led to a dramatic decrease in the expression of tRNAs (Fig. 5A). Consistent with previous observations after Pol II degradation in HEK293 cells [19] and with the results of triptolide treatment of mESCs followed by global run-on sequencing (GRO-Seq) analyses in mESCs [57], we observed that Pol II depletion caused both upregulation and downregulation of tRNAs (Fig. 5A, Additional file 8: Table S7). For example, Pol II depletion resulted in an increase in PRO-Seq signals at the tRNA regions upstream of Trim27 because of antisense transcription readthrough (Fig. 5B). The PRO-Seq signals for tRNA-Ala were decreased after Pol II depletion (Fig. 5C). Interestingly, we noted that Pol I depletion caused the dysregulated expression of several tRNAs (Fig. 5A), reminiscent of the enrichment of Pol III transcription in the perinucleolar compartment (PNC) reported previously [58]. The Pol II-affected tRNA genes were associated with preferentially active mRNAs or nearby Pol II ChIP-Seq peaks (Fig. 5D), a phenomenon that was also consistent with the local regulatory effect of Pol III on Pol II activity.

Analysis of differential chromatin accessibility at tRNA regions was performed, and the results showed that depletion of either Pol II or Pol III but not Pol I affected chromatin accessibility at tRNA regions (Fig. 5E). Moreover, tRNA genes downregulated upon Pol II depletion showed a greater increase in nucleosome occupancy than tRNA genes unaffected by Pol II depletion (Fig. 5F, Additional file 1: Fig. S3A, Additional file 9: Table S8). Previous reports have shown that Pol II depletion leads to repression of tRNA expression due to downregulation of C-MYC [19]. We also examined the protein levels of C-MYC and other transcriptional regulators after Pol II or Pol III depletion. Consistent with the aforementioned observation, Pol II depletion for 1 h slightly decreased the C-MYC protein level; in contrast, Pol III depletion did not obviously affect the level of C-MYC or that of any other regulators examined (Additional file 1: Fig. S3B). We then downloaded three independent C-MYC ChIP-Seq datasets from the ENCODE database (GSM288356, GSM3103385, GSM2417145) and found that C-MYC peaks did not overlap with down- and upregulated tRNAs after Pol II depletion (Additional file 1: Fig. S3C). These results indicated that the slight decrease in C-MYC may not fully explain the effects of tRNA expression after the loss of Pol II. On the other hand, it is possible that the chromatin binding of C-MYC at these tRNAs is weak or that these tRNAs might be less sensitive to the slight reduction in C-MYC due to the immediate degradation of Pol II. Together, these results suggest that the Pol II-mediated local nucleosome architecture may partially contribute to the transcription of specific tRNAs. However, we cannot completely rule out the possibility that C-MYC may directly or indirectly affect tRNA expression.

### Pol III depletion alters the Pol II interactome and significantly impairs FACT complex recruitment

**Fig. 6:**
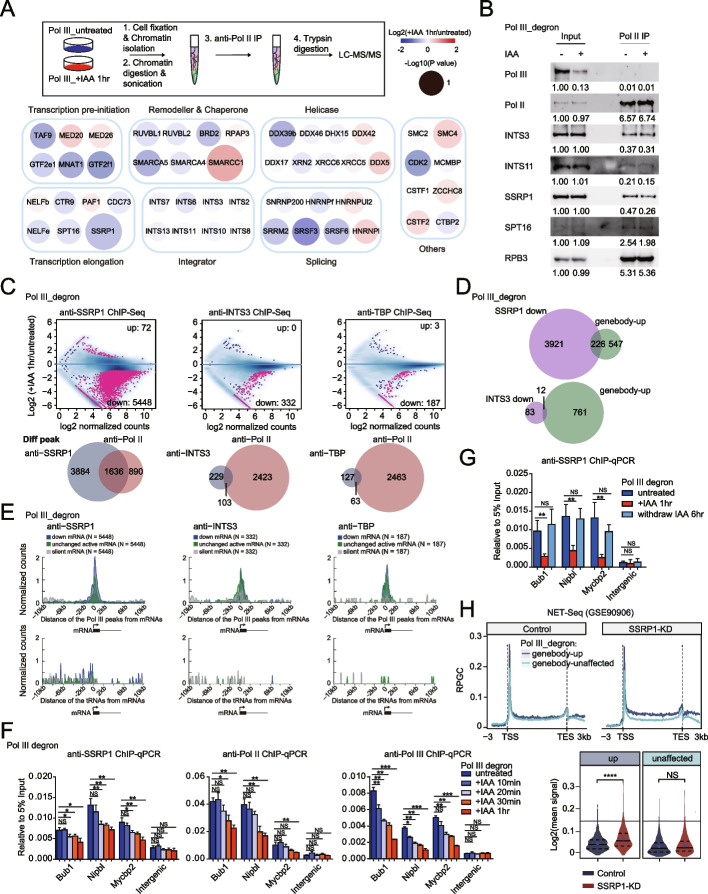
Pol III depletion alters the Pol II interactome and significantly impairs recruitment of the FACT complex. **A** Experimental setup for mass spectrometry of anti-Pol II immunoprecipitates obtained from crosslinking chromatin fractions of Pol III_degron cells that were untreated or treated with IAA for 1 h (upper). The protein factors listed here showed a similar trend of differential interactions with Pol II in both the canonical DDA and DIA-MS experiments (see the detailed procedure in the Supplementary Methods section). The color bar indicates proteins differentially interacting with Pol II, characterized by the log2FC and −log10(*P* value) with DIA-MS data (bottom). **B** Western blot analysis of anti-Pol II immunoprecipitates obtained from crosslinking chromatin fractions of Pol III_degron cells that were untreated or treated with IAA for 1 h revealed decreased interaction of FACT (SSRP1 and SPT16) and integrator (INTS3 and INTS11) complex components with Pol II after Pol III depletion. RPB3 is a subunit of Pol II with no change in its interaction with Pol II after the depletion of Pol III. The quantitative data shown under each panel were measured by ImageJ and normalized to the input under untreated conditions based on the representative figure shown. Three independent western blotting analyses were performed. Each showed a similar trend regarding a decreased interaction between FACT and Pol II upon Pol III depletion. However, the technical limitations of western blotting, such as large molecular weight or poor antibody sensitivity, may cause experimental variations. **C** MA plots showing differential enrichment of SSRP1, INTS3, and TBP ChIP-Seq signals in Pol III_degron cells under 1 h IAA treatment versus untreated conditions at Pol II peaks called in wild-type mESCs. Each dot represents one peak. Red indicates a significant change that meets both criteria of a false discovery rate (FDR) < 0.05 and fold change > 2 (upper). Venn diagrams comparing the Pol II-bound regions that had significantly altered binding affinities for SSRP1 (*N* = 5520, left), INTS3 (*N* = 332, middle), or TBP (*N* = 190, right) after Pol III depletion, as shown in the top panel, and the differential Pol II peaks upon Pol III depletion (*N* = 2526), as shown in Fig. 2D (bottom). **D** Venn diagrams showing the overlap of active promoters with significantly reduced SSRP1 (*N* = 4147, upper) or INTS3 (*N* = 95, lower) binding affinities with genebody-up mRNA genes upon Pol III depletion (*N* = 773). **E** Under the same representations described in Fig. 4A, downregulated mRNA genes upon Pol III depletion in SSRP1, INTS3, and TBP ChIP-Seq as identified in Fig. 6C were clustered based on their distance from the nearest Pol III-bound peaks and expressed tRNAs in 50 bp bins. Equal numbers of unchanged active genes and silent genes were selected as the control group. The *y*-axis in Fig. 6E represents the normalized counts of Pol III peaks or tRNAs by calculating their average density within 10 kb of the Pol II gene TSS for each category. **F** Bar graphs showing relative SSRP1, Pol II, and Pol III ChIP enrichment normalized to input (5%) at the presented example genes under Pol III untreated, + IAA 10 min, 20 min, 30 min, and 1 h conditions. Each sample was analyzed with two technical replicates per biological replicate and two biological replicates in total. Statistical significance was evaluated by Student’s *t* test (**: <0.01, *: <0.05). **G** Bar graphs showing relative SSRP1 ChIP enrichment normalized to input (5%) at the presented example genes under Pol III untreated, + IAA 1 h and +IAA 1 h followed by 6 h IAA withdrawal conditions. Each sample was analyzed with two technical replicates per biological replicate and two biological replicates in total. Statistical significance was evaluated by Student’s *t* test (**: <0.01, *: <0.05). **H** Under the same representations described in Fig. 4F, NET-seq data in mES cells were obtained from the public dataset (GSE90906). The average signal levels at the gene bodies using the gene groups from Fig. 4A were calculated and presented as metaplots and violin plots. The *p* value was calculated using the Mann–Whitney test

To gain further mechanistic insights into Pol III-mediated regulation of mRNA gene transcription, we performed Pol II ChIP-MS after Pol III depletion for 1 h; for these experiments, we prepared chromatin fractions to enrich the chromatin-associated factors (Fig. 6A). ChIP-MS captures many transient or nonspecifically interacting protein partners because of formaldehyde crosslinking, which means it can potentially capture the most targets. Thus, ChIP-MS data would provide a resource from which to identify candidate regulators. ChIP-MS was independently performed twice, once through data-dependent acquisition (DDA)-MS and once through data-independent acquisition (DIA)-MS. We next compared the differentially enriched proteins identified with DIA-MS with those identified through canonical DDA-MS, and the results showed an overlap of 37 downregulated and 15 upregulated proteins that interact with Pol II after Pol III depletion (Additional file 1: Fig. S3D, Additional file 10: Table S9, Additional file 11: Table S10). Several general transcription factors (GTFs), mediator subunits, PAF1, NELF, the integrator complex, and FACT components were affected in the Pol II interactome after Pol III depletion. GTFs and mediators are known to play roles in transcription initiation, and we wanted to investigate the roles of Pol III in regulating Pol II in the gene body regions. We found that the level of the PAF1 component CTR9 was decreased and that the PAF1 level was increased, and it was therefore difficult to find a consistent effect. Moreover, NELF was not linked to Pol III or chromatin structures as the references that we checked. Hence, we did not further investigate the aforementioned elongation factors. The levels of the FACT complex, which is known to regulate Pol II and Pol III [59–66] and to play roles in gene body transcription, appeared to decrease after Pol III depletion (Fig. 6A). These findings were further validated by RPB1 immunoprecipitation (IP) followed by western blotting analysis of Pol III-depleted cells (Fig. 6B). The quantitative decreases in the levels examined by western blotting were limited because Pol III depletion affects only a small fraction of Pol II-transcribed genes, so the changes in the Pol II interactome were likely lost in the background.

Then, ChIP-Seq to identify the FACT complex (SSRP1) and the integrator complex (INTS3) was performed after Pol III depletion; we used TBP, which is known to regulate transcription initiation for all three different Pols, as a control [21]. Differential analyses of the ChIP-Seq signals indicated a predominant decrease in chromatin binding for the FACT complex, but the reduction for that of the integrator complex and TBP binding was less dramatic (Fig. 6C). Interestingly, the sites of Pol II that were differentially affected after Pol III depletion overlapped substantially with those of FACT and overlapped less with those of the integrator complex and TBP (Fig. 6C). Furthermore, the genebody-up genes after Pol III depletion largely overlapped with the genes where FACT is affected by Pol III depletion and overlapped less with the genes that had defects in integrator complex binding (Fig. 6D). However, some genebody-up genes could not be explained by alterations of the FACT complex. The following reasons may account for the otherwise unexplained effects on genebody-up genes: many genebody-up genes may have exhibited decreases in FACT binding in the ChIP assay that were not significantly affected in the differential analysis due to the degree of change. Consistently, heatmap analyses indicated that the genebody-up genes exhibited decreased FACT binding (Additional file 1: Fig. S4A). Another explanation suggests that there are multiple mechanisms that may contribute to the Pol III regulation of Pol II activity, such as transcription interference and Pol III transcription of noncoding RNAs, as described previously. We also performed analyses of FACT-affected sites associated with Pol III binding sites and observed clear Pol III peak enrichment at FACT-affected sites but not at TBP- and INTS3-affected sites (Fig. 6E), which implies that Pol III binding at one site may locally regulate FACT recruitment at multiple sites in linear or spatial proximity. This phenomenon is similar to that of several genes that share the same enhancer, and multiple genomic regions may cluster in proximity to share regulators in the three-dimensional nucleus [67–69].

It is quite challenging to determine whether Pol III’s effects on the chromatin structure of nearby Pol II genes are a cause or consequence of Pol II transcription inhibition. We hypothesized that if Pol III affects Pol II transcription directly and the chromatin structure changes are the consequences, we would anticipate changes in Pol II chromatin occupancy ahead of that of the FACT complex. However, if Pol III affects chromatin structures and these changes affect transcription, then the chromatin binding changes of the FACT complex should be affected earlier than Pol II. To test this hypothesis, we performed SSRP1, Pol II, and Pol III ChIP–qPCR after acute Pol III depletion at different time points (untreated, 10 min, 20 min, 30 min, and 1 h of indole-3-acetic acid (IAA) treatment) to examine their chromatin binding at specific genes (Bub1, Nipbl and Mycbp2) (Fig. 6F). The results showed that the chromatin binding of Pol III immediately decreased after 10 min of IAA treatment, and FACT binding exhibited a significant decrease after 20 min of IAA treatment. In contrast, the Pol II distribution was significantly affected only after 30 min or 1 h of IAA treatment. In summary, the FACT response was evident earlier than the Pol II response after Pol III depletion. Furthermore, we performed SSRP1 ChIP–qPCR after Pol III depletion as well as its recovery, and we found that SSRP1 chromatin binding decreased after Pol III depletion and returned to normal levels when Pol III was recovered (Fig. 6G), suggesting that Pol III regulation of FACT chromatin occupancy was specific. In addition, we reanalyzed native elongating transcript sequencing (NET-Seq) data for a previously published FACT knockdown experiment, and the results showed that FACT knockdown preferentially caused an increase of Pol II-associated nascent RNA signals in gene body regions in genebody-up gene sets, similar to the trend after Pol III depletion (Fig. 6H). Collectively, these results support the idea that the FACT complex plays a crucial role in mediating the effects of Pol III on Pol II activity, underscoring alterations in nucleosome occupancy of nearby mRNA genes after Pol III depletion, as the FACT complex is known to directly interact with destabilized nucleosomes [70–73].

### Pol III depletion slows the Pol II transcription rate underpins FACT-mediated chromatin structure perturbations

**Fig. 7:**
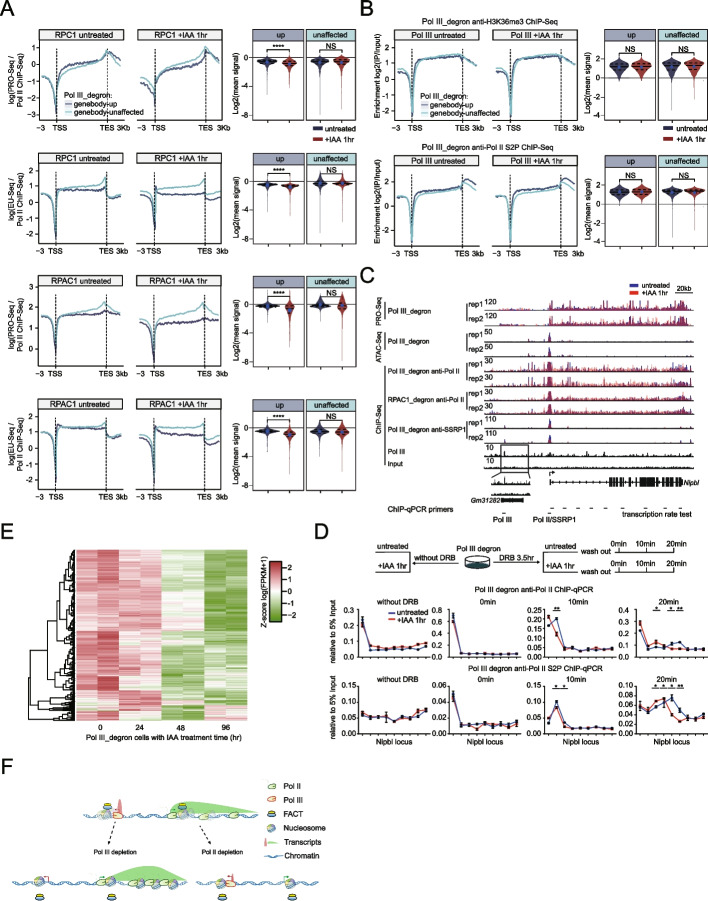
Pol III depletion increased nucleosome occupancy as an underlying mechanism related to slow down the Pol II transcription rate. **A** Mean coverage profiles for the ratio of PRO-Seq over Pol II ChIP-Seq signal (PRO-Seq/Pol II ChIP-Seq), as a proxy for transcription rate, and the ratio of EU-Seq over Pol II ChIP-Seq signal (EU-Seq/Pol II ChIP-Seq) upon RPC1 or RPAC1 depletion. Violin plots showing the quantification of the ratio changes for each group of gene sets in the right panel. The *p* value was calculated using the Mann–Whitney test. **B** Metaplots of H3K36me3 and S2P Pol II ChIP-Seq over the gene body regions of genebody-up mRNAs (*N* = 773, blue) and genebody-unaffected mRNAs (*N* = 3290, green) upon Pol III depletion. Violin plots showing the changes in ChIP-Seq signals over genebody-up or genebody-unaffected mRNA genes by Pol II upon depletion of Pol III, as shown in Fig. 3B (right). The *p* value was calculated using the Mann–Whitney test. **C** Genome browser track at the 8,269,424-8,592,237 region on chromosome 15 for Pol II ChIP-Seq signals in Pol III_degron and RPAC1_degron cells and SSRP1 ChIP-Seq signals in Pol III_degron cells that were untreated or treated with IAA for 1 h. PRO-Seq and ATAC-Seq in Pol III_degron cells that were untreated or treated with IAA for 1 h and Pol III ChIP-Seq in wild-type mESCs are shown in the same region. Only sense strand signals of PRO-Seq and ChAR-Seq are presented, and all tracks are flipped horizontally. **D** Upper panel: workflow of the DRB treatment assay with Pol III_degron cells that were untreated or treated with IAA for 1 h. Bottom panel: bar graphs showing relative ChIP enrichment normalized to input (5%) at the locus indicated in Fig. 7C. Each sample was analyzed with two technical replicates per biological replicate and two biological replicates in total. Statistical significance was evaluated by Student’s *t* test (**: <0.01, *: <0.05). **E** Heatmap representing the gene expression levels of the genebody-up genes upon Pol III depletion (*n*=773) from time-series RNA-Seq data (Pol III depletion for 0, 24, 48, and 96 h with two biological replicates). Each row represents the *z* score-transformed log2 (FPKM+1) values for one gene across different time points (green, low expression; red, high expression). **F** Working model for the cross-regulatory relationship between Pol II and Pol III by maintaining local chromatin structure. Pol II and Pol III help each other to destabilize nucleosome positioning and facilitate FACT-mediated chromatin structures maintenance when they occupy nearby regions in the genome

The increase in Pol II density on the gene body is consistent with the idea that FACT depletion reduces the rate of Pol II transcription. This supposition is consistent with a previous finding that FACT knockdown increased NET-Seq signals in mESCs [74]. We proposed that the PRO-Seq signals represent the nascent RNA synthesized during the nuclear run-on period and that Pol II ChIP-Seq indicates Pol II binding on chromatin. We then divided the PRO-Seq signals by the Pol II ChIP-Seq signals, yielding a ratio that should be correlated with the transcription rate. Specifically, the ratio of PRO-Seq/Pol II ChIP-Seq decreased for the genebody-up genes, but no obvious effects for the genebody-unaffected genes were observed upon Pol III depletion (Fig. 7A). We also observed a similar trend when measuring nascent RNA synthesis in cells by 5-ethynyluridine sequencing (EU-Seq) (Fig. 7A). To gain more information on the effects of Pol III depletion, we also performed H3K36me3 and Ser2 phosphorylated Pol II (S2P) ChIP-Seq. The results showed that Pol III depletion for 1 h did not obviously affect the abundance of H3K36me3 and S2P at either the genebody-up genes or the locus of a specific gene, such as Bub1 (Fig. 7B, Additional file 1: Fig. S4B). This finding differs from that of a previous report on FACT-mediated H3K36me3 modification in Drosophila because Drosophila genes are much shorter than mammalian genes [60], and therefore, transcription-coupled histone modifications may be more sensitive to FACT perturbation.

Furthermore, we first inhibited transcription with 5,6-dichlorobenzimidazole 1-β-D-ribofuranoside (DRB) for 3.5 h and released the transcription inhibition at different time points. Then, we collected samples for Pol II and S2P Pol II ChIP–qPCR analyses to determine the transcription rate of the long active genes Nipbl and Mycbp2 before and after Pol III (RPC1) depletion. The ChIP–qPCR results indeed showed that Pol III depletion reduced the Pol II transcription rate (Fig. 7C,D, Additional file 1: Fig. S4C-D, Additional file 12: Table S11). In addition, two independent replicates of the dataset showed a decrease in SSRP1 binding and chromatin accessibility and increased Pol II occupancy at the gene bodies (Fig. 7C, Additional file 1: Fig. S4C and S4E), and read per genome coverage (RPGC) normalized count quantification was significantly increased in the gene body regions of Pol II ChIP-Seq and PRO-Seq and was decreased in the promoter regions of ATAC-Seq and SSRP1 ChIP-Seq upon Pol III or RPAC1 depletion at the Nipbl and Mycbp2 regions (Additional file 1: Fig. S4E). We next performed polyA RNA-Seq after Pol III depletion for 24, 48, and 96 h. A heatmap was prepared and showed that after Pol III depletion, the genebody-up genes showed a gradual decrease in mRNA expression levels by time-course RNA-Seq (Fig. 7E). The expression of a subset of genes did not decrease, perhaps because of the secondary effects after long-term depletion of Pol III. We next plotted the EU-seq signal in genebody-up and genebody-unaffected gene sets. Both the Pol III degron and its small subunit RPAC1 degron significantly decreased EU-seq signals in these genebody-up gene sets (Additional file 1: Fig. S4F). These results collectively indicated that Pol III depletion reduced the Pol II transcription rate, thereby decreasing the levels of mature transcripts.

## Discussion

The transcriptional activities of Pol I, Pol II, and Pol III were identified decades ago. However, it has not been systematically elucidated that the genes in the mammalian genome are regulated by specific Pols. To identify these, we identified the binding sites for each Pol and disrupted the expression of individual Pols to examine the effects on the other two Pols. By combining multiomics analyses and acute protein degradation techniques, we found that Pol I, Pol II, and Pol III predominantly transcribe specific genes, with only a few instances of cross-regulation. One of the most prominent cross-regulatory effects of Pols is that Pol III preferentially regulates Pol II transcription at nearby sites (*n*=773 mRNA genes). Pol III was found to be required for the maintenance of local chromatin architecture, recruitment of the FACT complex, and the Pol II transcription rate for nearby mRNA genes (Fig. 7F). This local chromatin architecture-based mechanism also helps to explain the effects of Pol II on Pol III-mediated tRNA transcription (Fig. 7F). Our results offer new insights into the reliance of Pol cross-regulation on the active chromatin architecture and provide a foundation to further investigate the roles of different Pols during development and diseases.

ChIP-Seq or transcription inhibitors are usually used to identify the target genes of Pols [14–18, 75, 76]. ChIP-Seq identified the chromatin binding sites of the Pols but did not confirm that they were functional. In addition, Pols-specific interacting protein partners or noncoding RNAs were investigated to determine whether they mediate the cross-regulatory relationship between different Pols. However, these studies could not rule out the possibility that these factors function independently of Pols, and therefore, these studies did not reveal the direct cross-regulatory relationships between different Pols. In this study, we first identified Pol I, Pol II, and Pol III binding sites in the genome and then rapidly degraded one Pol (for 1 h) and performed ChIP-Seq for the other Pols as well as nascent RNA sequencing. This strategy allowed us to systematically identify genes specifically regulated and cross-regulated by Pol I, Pol II, and Pol III genome-wide.

Increasing evidence has shown the close association between Pol II and Pol III throughout the genome [14–17]. Cross-regulatory relationships between different Pols have been reported at specific genes or for specific regulatory types [18–20, 22, 50, 76]. The comprehensive cross-regulome of Pol I, Pol II, and Pol III, however, has not been investigated to date. Excitingly, we indeed identified multiple known cross-regulated gene loci and more genes that have not been reported to be cross-regulated by different Pols. Through orthogonal experimental analyses, we found that most of these genes were transcribed by specific Pols without noticeable cross-regulation by different Pols. The genomic binding sites of only two RNA polymerases were located near each other in either the 1D or 3D structures, and these sites may be cross-regulated, at least by Pol II and Pol III. On the other hand, the magnitude of the cross-regulation is not very robust, and it appears to fine-tune gene expression. However, we could not rule out the possibility that these regulatory effects may be essential for cells undergoing dramatic environmental changes, such as serum starvation or cell cycle progression.

Previous studies have shown that FACT recognizes destabilized nucleosomes in chromatin [70, 77–80] and plays key roles in transcriptional regulation. Our ATAC-Seq analyses indicated the increased occupancy of nucleosomes after acute depletion of Pol III. Consistently, we observed decreased binding for the FACT complex and a slowdown of the Pol II transcription rate. Furthermore, we also analyzed the previously published NET-Seq data after the knockdown of the FACT complex in mESCs and showed that our genebody-up genes also retarded Pol II transcription signals after FACT knockdown. These results suggested that Pol III regulates the Pol II transcription rate, the recruitment of FACT, and the structure of local chromatin. We could not completely rule out the possibility that the decrease in Pol II binding to the promoters induces a reduction in FACT recruitment [72]. However, we do not think this model conflicts with our model, and we believe that both mechanisms are needed to fully explain the defects in Pol II transcription after Pol III depletion. Nucleosome unwrapping was impaired nearby, and FACT recruitment was decreased after Pol III depletion. The decrease in Pol II binding at the promoter-proximal regions was confirmed by time-series ChIP–qPCR at specific gene loci. Then, these decreased Pol II occupancies would further decrease FACT recruitment to the +1 nucleosome. This positive feedback relationship between Pol II and FACT causes massive defects in FACT recruitment and Pol II occupancy, as observed in our Pol II and FACT ChIP-Seq datasets.

Previous studies have revealed multiple mechanisms of cross-regulation between different Pols [18–20, 26, 27, 50], such as transcriptional interference, chromatin looping, and noncoding RNAs. We carefully categorized the Pol III-affected Pol II genes into subgroups based on different features (Fig. 4B), and we hypothesized that multiple models help to explain the cross-regulatory relationship between Pol III and Pol II. It is possible that Pol III regulates Pol II through different independent and combinatory mechanisms at different genes.

How Pol III regulates the nucleosome positioning near Pol II genes is unknown; we speculate that Pol III transcription leads to a highly dynamic histone eviction and deposition, and such histone exchange may serve as a molecular reservoir of histones, chromatin remodelers, and molecular chaperones that influence the histone dynamics of nearby Pol II genes. This was the reason why we observed that Pol III depletion increased nucleosome positioning at the TSSs of nearby Pol II genes. On the other hand, the histone dynamics at Pol II genes also provide positive feedback to the histone exchange process occurring at nearby Pol III genes, it comports with our findings showing that Pol II depletion also increased nucleosome positioning at nearby Pol III genes. Consequently, our results implicate mutually beneficial histone dynamics at adjacent Pol II and Pol III genes.

The proximity between Pol III binding sites and nearby mRNA genes, and this regulatory relationship may be conserved across different species. This possibility provides insights for investigating the biological functions of Pol III-transcribed tRNAs or retrotransposon elements in other species, which are worthy of further investigation. On the other hand, the 3D proximity between bound Pol III and its interacting mRNA genes, achieved through chromatin looping, may be more dynamically regulated, which would impart functional diversity to Pol III in various biological systems to fine-tune the expression of different mRNAs.

## Conclusions

Herein, we provide a perspective on the cross-regulatory effects of different Pols. We found that although they mainly affect their own target genes, some genes otherwise transcribed by different Pols are mutually regulated. Strikingly, the main crosstalk mechanism is exemplified by the fact that Pol III depletion affects Pol II transcription. Pol III functions as a regulator of the FACT-Pol II axis to alter local chromatin structures, which affects the Pol II transcriptional rate at some gene sites with diverse cellular functions. These results help us understand the dysregulation of Pol III in developmental diseases of various tissues.

## Methods

### Key resource table

**Table:** 

Reagent or resource	Source	Identifier
Antibodies		
Anti-POLR1A	Santa Cruz	Cat # Sc-48385
Anti-RNA Pol II-CTD	Abcam	Cat # ab817
Anti-RNA Pol II-NTD	CST	Cat # D8L4Y
Anti-POLR3A	Abcam	Cat # ab96328
Anti-GFP	Abcam	Cat # ab290
Anti-HA tag	Abcam	Cat # ab9110
Anti-β-Actin	Sigma-Aldrich	Cat # A2228
Anti-RPAC1	Santa Cruz	Cat # Sc-374443
Anti-SSRP1	Biolegend	Cat # 609710
Anti-SUPT16H	Santa Cruz	Cat # Sc-165987
Anti-INTS3	Proteintech	Cat # 16620-1-AP
Anti-INTS11	ABclonal	Cat # A6566
Anti-TBP	Proteintech	Cat # 22006-1-AP
Anti-RNA Pol II S2p	CST	Cat # 13499
Anti-H3K36me3	Abcam	Cat # Ab9050
Anti-C-MYC	Proteintech	Cat # 10828-1-AP
Anti-RPB3	Proteintech	Cat # 13428-1-AP
Anti-RPB5	Proteintech	Cat # 15217-1-AP
Anti-RPB6	Proteintech	Cat # 15334-1-AP
Anti-RPB8	Proteintech	Cat # 15086-1-AP
Anti-RPB11	Proteintech	Cat # 16403-1-AP
Anti-RPAC1	Proteintech	Cat # 15923-1-AP
Anti-Rabbit IgG Secondary Antibody	GE Healthcare	Cat # NA931V
Anti-Mouse IgG Secondary Antibody	GE Healthcare	Cat # NXA931V
Chemicals		
Normal Mouse IgG	Merck Millipore	Cat # 12-370
Normal Rabbit IgG	Merck Millipore	Cat # 12-370
Complete Tablets EDTA-free, EASYpack	Roche	Cat # 04693132001
Doxycycline	Sigma-Aldrich	Cat # D9891
Indole-3-acetic acid sodium salt	Sigma-Aldrich	Cat # Cat # 1I5148
Puromycin dihydrochloride	Sigma-Aldrich	Cat # P8833
GENETICIN, G418	Thermo Fisher	Cat # 10131035
5-ethynyluridine (EU)	J&K	Cat # 1388360
Biotin-PEG3-azide	Aladdin	Cat # B122225
THPTA	Sigma	Cat # 762342
Sodium ascorbate	Sigma	Cat # A7631
Critical commercial assays		
Dynabeads™ Protein G	Thermo Fisher	Cat # 10004D
Dynabeads™ M-280 Streptavidin	Thermo Fisher	Cat # 11205D
Pierce^TM^ BCA protein assay kit	Thermo Fisher	Cat # 23227
FuGENE® HD Transfection Reagent	Promega	Cat # E2311
NEBNext Ultra II DNA Library Prep Kit for Illumina	NEB	Cat # E7645S
Illumina Nextera DNA Sample Preparation Kit	Illumina	Cat # FC-121-1030
KOD FX polymerase	TOYOBO	Cat # KFX-101
2×Taq Master Mix (Dye Plus)	Vazyme	Cat # P112-02
pEASY-Basic Seamless Cloning and Assembly Kit	TransGen	CU201-03
KAPA HIFI hotstart PCR Kit	Kapa Biosystems	Cat # KK2502
SuperScript™ III Reverse Transcriptase	Thermo Fisher	Cat # 18080085
P-30 RNase-free spin column	BioRad	Cat # 732-6250
Micrococcal Nuclease (MNase)	NEB	Cat # M0247S
ChamQ Universal SYBR qPCR Master Mix	Vazyme	Cat # Q711-02
Megen Gel extraction kit	Megen	Cat # D2111-03
HiPure Plasmid EF Mini Kit	Magen	Cat # P1112-02
Qubit dsDNA HS kit	Thermo Fisher	Cat # Q32851

### Mouse ES cell culture

The V6.5 mouse ES (mES) cell line used here was a gift from R. Young of the Whitehead Institute, which was derived from the inner cell mass (ICM) of C57BL/6 × 129/sv crossed mice. These mES cells were cultured as previously described [38] and were tested and found to be free of mycoplasma contamination every 3 months. For experiments, all degron mES cells were pretreated with 1 μg/ml doxycycline for 12 h and then were treated with or without 500 μM indole-3-acetic acid (IAA) for different time points.

### Plasmid construction and gene targeting

Plasmids for degron cell line construction are constructed as described [38]. Briefly, gene targeting donors contain mAID-GFP tag flanked with mouse genomic sequence for targeted loci were constructed with seamless ligation kit (TransGen Biotech, Cat # CU201-03). The donor of RPA1, RPB1, RPC1, and RPAC1 are fused to their C-terminal domain of endogenous loci, respectively. For comparison, we insert a mAID-GFP tag into RPB1 N-terminal domain to construct Pol II_NTD_degron cell line. For cell transfection, plasmids of donor and CRISPR sgRNA were prepared using a HiPure Plasmid EF Mini Kit (Magen, Cat # P1112-02) and were transfected into Tir1 stable-expressing clonal mouse embryonic stem cell line using FuGENE HD (Promega, Cat # E2311) following the manufacturer’s protocol. After 2 days, the cells were passaged and grown for 1 week in the presence of 100 μg/ml neomycin in the medium. The homozygous clonal lines were selected after genotyping. These clonal lines were assessed for their ability to undergo IAA-induced degradation and to show expression levels similar to that in wild-type mES cells. The clones degraded with the maximum efficiency were chosen for the following assays.

### Western blotting

mESCs were dissociated, centrifugated at 3500 rpm for 2 min to be pelleted, and resuspended in 2.5 mM MgCl_2_, 0.1% NP40, 0.25 M sucrose, 1 mM DTT, 700 mM NaCl, 25 mM HEPES pH 7.9, 1× protease inhibitor cocktail and lysis for 10 min on ice, centrifugated at 14,000 rpm for 10 min at 4 °C. The protein concentration of supernatants was measured using the PierceTM BCA protein assay kit (Thermo, Cat # 23227). Samples were mixed with 2×loading buffer (4% SDS, 20% Glycerol, 0.2 M DTT, 0.2% Bromophenol Blue) for 10 min at 100 °C. Samples run on 10–12% polyacrylamide SDS-PAGE gel and transfer onto PVDF membranes were performed with 300 mA for 2 h. Membranes were blocked with 5% skim milk in PBST for 1 h at room temperature and then incubated with primary antibody diluted in 5% bovine serum albumin of PBST following the manufacturer’s recommendation overnight at 4 °C. The next day, the membrane was washed three times 5 min in PBS-0.1% Tween-20 at room temperature, incubated with secondary antibodies (1: 10,000) in 5% bovine serum albumin in PBS buffer supplementing with 0.1% Tween-20 1 h at room temperature, washed 3 times, and analyzed on G.E AI 600 RGB imaging system. Panels were mounted using ImageJ preserving linearity.

### Native chromatin fraction isolation for immunoprecipitation

mESCs were dissociated, centrifugated at 3500 rpm for 2 min to be pelleted, and washed once with PBS/1 mM EDTA. mESCs were resuspended in CE buffer (10 mM HEPES pH 7.6, 60 mM KCl, 1 mM EDTA, 0.1% NP40, 1 mM DTT, 0.34 M Sucrose) on ice for 5 min and centrifugated at 3500*g* for 15 min at 4 °C, supernatant (cytoplasm) was discarded, and pellets were washed once with PBS/1 mM EDTA. The pellets were resuspended in glycerol buffer (20 mM Tris·HCl pH 8.0, 75 mM NaCl, 0.5 mM EDTA, 0.85 mM DTT, 50% (vol/vol) glycerol) following an equal volume of nuclei lysis buffer (10 mM Hepes pH 7.6, 1 mM DTT, 7.5 mM MgCl_2_, 0.2 mM EDTA, 0.3 M NaCl, 1 M urea, 1% NP-40), mixed thoroughly and placed on ice for 2 min, and centrifugated at 12,000 rpm for 2 min at 4 °C, and the supernatant (nucleoplasm) was discarded. The pellets (chromatin fraction) were washed twice with 1 ml PBS/1 mM EDTA. Chromatin in CSKII buffer was resuspended (10 mM PIPES pH 6.8, 50 mM NaCl, 0.3 M Sucrose, 6 mM MgCl, 1 mM DTT) with 5 μl DNase I (NEB, Cat # M0303) and 5 μl RNase A (Takara, Cat # 2158), 700 rpm rotation at 37 °C for 30 min. Equal volume of CSKII+ 0.5 M (NH4)2SO4 buffer was added and incubated at room temperature for 10 min. The mixture was centrifugated at 1200*g* for 6 min, and the supernatant fractions were collected. After incubating with antibody and protein G coupled beads for 12 h at 4 °C, the beads were washed four times with wash buffer (30 mM Hepes pH 7.6, 0.1 M NaCl, 0.5% NP-40 and proteinase inhibitor) and boiled at 100 °C for 10 min. Supernatants were collected for western blot detection.

### ATAC-Seq

ATAC-Seq was performed according to the protocol from [81]. In total, 50,000 viable cells were used for library preparation using Nextera™ DNA Sample Prep Kit (Illumina, Cat # FC-121-1030). PCR-amplified libraries were extracted with Megen gel purification kit (Megen, Cat # D2111-03) without size selection. Library quality and quantity were analyzed with Bioanalyzer and Qubit assays and then sequenced on Illumina HiSeqXten using 150 bp paired-end mode.

### ChIP-Seq/qPCR, ChIP-mass spectrometry

The ChIP procedure was modified based on a previously published protocol [82]. After cell fixation with 1% formaldehyde for 10 min at room temperature, chromatin fractions were isolated and 40 U of micrococcal nuclease (NEB, Cat # M0247S) was added to the chromatin fraction, incubated at 37 °C for 15 min, and then added 20 μl 0.5 M EDTA and 40 μl 0.5 M EGTA to inactivate MNase. The spun-down pellets were resuspended in 1 ml of sonication buffer and were sonicated by a Biorupter with the following settings: high energy, 30 s working time, 60-s intervals, 20 cycles. After centrifugated twice at 12,000 rpm for 10 min at 4 °C, the subsequent antibody enrichment and ChIP DNA/protein collection procedures were conducted as previously described. For ChIP-Seq library construction, all the ChIP material or 20 ng of the input ChIP DNA was used to construct Illumina sequencing libraries using the NEBNext Ultra II DNA Library Prep Kit for Illumina (NEB, Cat # E7645S). PCR-amplified libraries were gel extracted at 200–500 bp and eluted in 30 μl of water. The library quality and quantity were analyzed with Bioanalyzer and Qubit assays, and then, the library was sequenced using HiseqXten 150 × 150 pair-end sequencing. For ChIP-qPCR, we used primers with ChamQ Universal SYBR qPCR Master Mix (Vazyme, Cat # Q711-02). For ChIP-WB detection, the samples were detected as the “Western blotting” section described. For ChIP-MS, the samples were loaded for high-resolution MS detection (Thermo Fisher, Orbitrap Fusion Lumos) under the manufacturer’s instruction.

### DRB treatment ChIP-qPCR

Pol III_degron cells were added with 1 μg/ml doxycycline for 12 h. For DRB treatment groups, 100 μM DRB was added for 3.5 h. For DRB release assay, DRB were washed out twice with PBS and replaced with fresh medium for 0, 10, and 20 min, and formaldehyde fixation was conducted immediately as aforementioned. For Pol III degradation groups, 500 μM indole-3-acetic acid (IAA) was added when cell was treated with DRB for 2.5 h so that cell was treated with 3.5 h DRB and 1 h IAA when harvested, and doxycycline and IAA were maintained in the medium in DRB release process. ChIP-qPCR were conducted as aforementioned.

### Chromatin-associated RNA-Seq

The cells were dissociated and counts were 2×10^7^ for one experiment. After adding 5% Drosophila S2 cells as spike-in, the mixed cell population was pelleted by centrifugation at 1000*g* for 5 min at 4 °C. Cell pellets were lysed gently with 0.5 ml of ice-cold NP-40 lysis buffer (10 mM Tris·HCl pH 7.5, 150 mM NaCl, 0.05% NP-40) on ice for 5 min. The cell lysate was added on top of 1.25 ml sucrose cushion (24% sucrose (wt/vol) in NP-40 lysis buffer). Centrifugation was done at 12,000 rpm for 10 min at 4 °C to isolate the nuclei pellet (the supernatant represented the cytoplasmic fraction). The nuclei pellet was washed once with 1 ml PBS/1 mM EDTA. Centrifugation was done at 12,000 rpm for 1 min at 4 °C, and the supernatant was discarded. Then, 0.5 ml nuclei lysis buffer (10 mM Hepes pH 7.6, 1 mM DTT, 7.5 mM MgCl_2_, 0.2 mM EDTA, 0.3 M NaCl, 1 M urea, 1% NP-40) and 0.5 ml glycerol buffer (20 mM Tris·HCl pH 8.0, 75 mM NaCl, 0.5 mM EDTA, 0.85 mM DTT, 50% (vol/vol) glycerol) were mixed. The nuclei pellet was resuspended gently and incubated on ice for 2 min. Centrifugation was done at 12,000 rpm for 2 min at 4 °C, and the supernatant was discarded (The supernatant represented the nuclear soluble fraction). The chromatin pellet was washed twice with 1 ml PBS/1 mM EDTA. Centrifugation was done at 12,000 rpm for 1 min at 4 °C, and the supernatant was discarded. Total RNA was isolated using TRIzol following the manufacturer’s instructions. Sequencing libraries were generated by Novogene corporation. The libraries were sequenced on an Illumina HiseqXten platform, and 150 bp paired-end reads were generated.

### PRO-Seq

PRO-Seq was modified based on a previously published protocol [83]. mES cells were dissociated and counts were ~10^7^ for one experiment. After adding 5% Drosophila S2 cells as spike-in, the mixed cell population was pelleted by centrifugation at 1000*g* for 5 min at 4 °C. The cell pellet was washed once in 10 ml of ice-cold PBS and resuspended in ice-cold douncing buffer (1×10^6^ cells per ml, 10 mM Tris-HCl pH 7.4, 300 mM sucrose, 3 mM CaCl_2_, 2 mM MgCl_2_, 0.1% (vol/vol) Triton X-100, 0.5 mM DTT) for 5 min on ice and dounced 25 times using a Dounce homogenizer, followed by washing twice with douncing buffer. After centrifugation, the pellet was resuspended in storage buffer (5–10 × 10^6^ nuclei per 100 μl of storage buffer, 10 mM Tris-HCl pH 8.0, 25% (vol/vol) glycerol, 5 mM MgCl_2_, 0.1 mM EDTA, and 5 mM DTT), and the solution was moved forward or flash-frozen in liquid nitrogen and stored at −80 °C. A 100 μl 2× NRO master mix was prepared (10 mM Tris-HCl pH 8.0, 5 mM MgCl_2_, 1 mM DTT, 300 mM KCl, 0.02 mM biotin-11-CTP, 0.0005 mM CTP, 0.25 mM ATP/GTP/UTP, 1% Sarkosyl and RNase inhibitor), pipetted thoroughly, and preheated to 37 °C. Using a cutoff P200 pipette tip, 100 μl of nuclei was added gently but the mixture was thoroughly pipetted 15 times and the cells were incubated for 3 min, with gently tapping at the incubation midpoint. Then total RNA was extracted with Trizol LS and dissolved with 20 μl DEPC-H_2_O. RNA was heat-denatured at 65 °C on a heat block for 40 s and fragmented by base hydrolysis by adding 5 μl of ice-cold 1 M NaOH, and the mixture was incubated on ice for 10 min. After adding 25 μl of 1 M Tris-HCl, pH 6.8, buffer was exchanged once by running the 50 μl base-hydrolyzed RNA sample through a P-30 RNase-free spin column according to the manufacturer’s instructions (BioRad, #732-6250). Then, ~50 μl of the RNA sample from the prior step and 50 μl of prewashed M280-streptavidin beads were mixed and incubated at room temperature on a rotator for 20 min. After washing beads with ice-cold high-salt wash buffer (50 mM Tris-HCl pH 7.4, 2 M NaCl and 0.5% (vol/vol) Triton X-100), binding buffer (10 mM Tris-HCl pH 7.4, 300 mM NaCl and 0.1% (vol/vol) Triton X-100), and low-salt wash buffer (5 mM Tris-HCl pH 7.4 and 0.1% (vol/vol) Triton X-100) each two times, RNA was extracted with Trizol twice and precipitated with glycogen and dissolved with 16 μl DEPC-H_2_O. RNA reverse transcription was conducted with SuperScript™ III Reverse Transcriptase according to instruction (Thermo Fisher, Cat # 18080085). After eliminating RNA by adding 2 μl of 1 M NaOH and incubating 20 min at 98 °C, the single-strand DNA was used to construct library with TELP protocol [3]. PCR-amplified libraries were gel extracted at 200–500 bp and eluted in 30 μl of water. The library quality and quantity were analyzed with Bioanalyzer and Qubit assays. The library was sequenced using HiseqXten 150 × 150 pair-end sequencing.

### EU-Seq

EU-Seq was modified based on a previously published protocol [84]. Briefly, the cell-permeable uridine analog, 5-ethynyluridine (EU), is added to the culture medium with 1 mM concentration for 10 min to allow in vivo labeling of nascent transcripts. Ten percent of the total cell number of Drosophila S2 cells were treated similarly and used as spike-in control. After EU labeling, the cells are lysed, and total RNA is extracted. Biotin is conjugated to 10 μg EU-labeled RNAs with a click chemistry reaction in 30 μl working solution (50 mM HEPPS-pH 7.5, 2.5 mM THPTA, 2.5 mM CuSO_4_, 4 mM Biotin-PEG_3_-azide, 10 mM sodium ascorbate) for 1 h at room temperature. The reaction is stopped with 450 μl 5 mM EDTA and then the biotinylated RNAs are extracted with 500 μl phenol-chloroform (pH 5.2). The supernatant is collected by centrifugation at 13,000 rpm 4 °C for 10 min. 1/10 volume of 3 M NaAC, 1 μl glycogen, and an equal volume of Isopropanol are added to the supernatant. The RNAs are precipitated by centrifugation at 13,000 rpm 4 °C for 20 min, washed once with 75% EtOH, and dissolved in 100 μl H_2_O. Biotin-labeled RNAs were hydrolyzed with NaOH and subsequently enriched by streptavidin beads. cDNA synthesis and library construction were conducted as PRO-Seq described. PCR-amplified libraries were gel extracted at 200–500 bp and eluted in 30 μl H_2_O. The library quality and quantity were analyzed with Bioanalyzer and Qubit assays. The library was sequenced using HiseqXten 150 × 150 pair-end sequencing.

### ChIP-Seq mapping and analysis

ChIP-Seq raw data were processed as described [38]. Adapters were trimmed with cutadapt (v2.10) in paired-end mode with the following parameters: -q 15,15 –minimum-length 18 -a CCCCCCCCCAGATCGGAAGAGCACACGTCTGAACTCCAGTCAC -A AGATCGGAAGAGCGTCGTGTAGGGAAAGAGTGT. Trimmed reads were first aligned to the mouse reference genome (mm10) using Bowtie2 (v2.3.5.1) with default options [85]. SAMtools (v 0.1.19) and sambamba (v0.7.0) were used to filter unmapped reads, multiple mapped reads, and potential PCR duplicates [4, 5]. Only uniquely aligned reads were retained before calling peaks with MACS2 (v2.2.5) [6]. Publically available ChIP-Seq data from mESCs (Additional file 2: Table S1) were processed using the same strategy. For visualization, bam files of individual replicates for each condition were combined and then converted to bigwig files, binned (10 bp), and normalized to 1× depth of reads per genome coverage (RPGC) using the bamCoverage from the deepTools suite (v3.4.3) with parameters “–normalizeUsingRPGC –bs 10” [86]. Downstream analyses such as TSS plots, metagene plots, and heatmaps were also performed using deepTools with a bin size of 50 bp. Peaks were annotated within mRNA or lncRNA regions according to the GENCODE definitions, while the repeat elements (such as SINE, LINE, or LTR) were provided by Homer software (v4.10) [87], and tRNA loci are available on the UCSC Table Browser. Peaks were ensured to fall only within one single category. Annotation of shared and specific Pol I, Pol II, and Pol III binding peaks was carried out using bedtools combined with the Homer annotatePeaks.pl script, then visualized as a pie or bar graph using ggplot2 R package. The wide-type RNAP peaks were downloaded from supplementary data of our previous study. DiffBind (v.2.14.0) was used for the differential analysis of indicated ChIP-Seq signals by perturbating another two RNA polymerase at wide-type peaks of itself or active promoters [88] (defined in the “Gene list and promoter definition” section).

### ChIP-Seq mapping to rDNA units

Trimmed reads were independently aligned against a single copy of the mouse rDNA repeat sequence (GenBank: BK000964.3) using bowtie2 to study whether Pol II and Pol III disruption exert an active or repressive effect on rDNA transcription level. The bam files from sample replicates were then merged to create a representative genome track over the rRNA gene unit, as shown in Fig. 2A.

### Poly(A) RNA-Seq mapping and analysis

Raw data were processed as previously described [38]. After quality control and ribosomal read removal, adapter sequences were removed and aligned against the concatenated genome of mm10 and dm6 using the STAR aligner (v 2.7.5a) with default parameters and then separated into the mouse and fly bins [89]. The uniquely mapped reads were counted to estimate the transcript abundance over the Gencode annotated genes (mm10, GRCm38/M23) using featureCounts (v2.0.1) [90]. Differentially expressed genes were detected using DESeq2 with significance cutoffs of FDR < 0.05 and fold change > 2, with a minimum read count of 1 in at least one control sample of two biological replicates [91].

### ATAC-Seq mapping and analysis

ATAC-Seq data were processed as published. Briefly, sequenced reads were first adapter trimmed and quality verified with cutadapt and FastQC (v0.11.7). Clean reads were then aligned to the mm10 mouse genome using Bowtie2. Uniquely aligned reads were subsequently processed into sorted, indexed BAM files using SAMtools. PCR duplicates and mitochondrial reads were discarded before further analysis. The remaining reads were corrected to account for the 9-bp insert introduced by the Tn5 transposase by offsetting the 5′ ends by either +4 (for plus strand) or −5 (for minus strand) as described previously. RPGC-normalized bigwig tracks representing open chromatin accessibility were generated using deepTools bamCoverage with parameters “–normalizeUsingRPGC –bs 10.” Nucleosome positioning and the corresponding signal tracks were calculated from replicate-merged ATAC-Seq data using the nucleoATAC algorithm (v0.3.4) with default parameters [92].

### Gene list and promoter definition

All gene-centric analyses in this study were performed using mouse GENCODE annotation downloaded from gencodegenes.org in GTF format and filtered such that only “gene” entries. Annotations from chrM and random chromosomes were also omitted.

Active genes were defined as having promoter-proximal density is greater than 0, and the gene body density is significantly higher than 0.04 reads/kb based on the background estimation in our untreated PRO-Seq data, as previously described [93]. A union list of 8845 active mRNA genes (supported by RefSeq annotation) was created by only retaining those detected from all three untreated samples in mESCs and with a minimum length of 5 kb. The coordinates and annotations of tRNAs were downloaded in BED format from the UCSC Table Browser. Intronless genes were also extracted from RefSeq annotated genes comprising one single isoform with one exon.

Promoter regions were identified by ±1 kb regions surrounding the annotated transcription start sites. Active promoters were those promoters of active genes and overlapped with the H3K4me3 peak.

### PRO-Seq and chromatin-associated RNA-Seq (ChAR-Seq) mapping and analysis

PRO-Seq and ChAR-Seq data were processed using a custom pipeline that builds on published workflows with minor modifications. Ribosomal reads were first removed by mapping to one copy of the mouse rDNA sequence. After adapter trimming and quality control with cutadapt and RseQC (v4.0.0) separately, clean reads were then aligned to the concatenated mm10+dm6 genome using bowtie2 with default options [94].

Nonuniquely mapping or properly paired reads were discarded, and PCR duplicates were removed with Sambamba. Read mapping to mouse and Drosophila chromosomes were separated and counted with SAMtools. Then, to quantitatively compare gene expression and genome enrichment profiles between different conditions or perturbations, the PRO-Seq and ChAR-Seq data were internally calibrated with Drosophila spike-in cells as previously introduced [38]. featureCounts was used to get gene-level read counts from uniquely mapped bam files in a strand-specific manner. This quantification procedure includes signals only in the gene body (+300 bp from TSS to annotated gene end), while very lowly expressed genes with less than five reads in all samples were also excluded from subsequent analysis. The resultant gene read count table was then subjected to DESeq2 for differential expression analysis, and a cutoff of 0.05 for FDR was chosen to identify significantly differential genes.

For visualization, BAM files of biological replicates were highly correlated and were pooled together before converting to bigwig signal tracks. The final bigwig files were separated by strand and normalized to spike-in controls using bamCoverage from deepTools with a bin size of 10. Pausing index was calculated according to the previous report [93, 95], defined as the read coverage in the gene body (from TSS+300 bp to the gene end) over the promoter-proximal region (from −30 to +300 bp relative to the TSS) for each gene. Only genes with a minimum length of 5 kb were considered in this analysis.

### BETA analysis to combine ChIP-Seq and PRO-Seq results

We associated Pol III binding regions with nearby Pol II genes using Binding and Expression Target Analysis (BETA) (v1.0.7) to predict whether Pol III has an activating or repressive function by combining ChIP-Seq and PRO-Seq results [96]. The analysis was performed as previously published with the following adaptations. To study the direct functions of Pol III on Pol II-transcribed mRNA genes, we propose two types of interaction models: spatial proximity based on H3K27ac HiChIP loops (obtained from the previous study) and local regulation according to nearest mRNA genes. Briefly, each Pol III peak was independently classified according to its overlap profile, following a hierarchical tree. That is, we first assigned the Pol III peak to H3K27ac HiChIP contact anchors (requiring a minimum 1-bp overlap) to find its mRNA partner on the other side, while the associated target genes within ±100 kb of unassigned Pol III peaks were identified by linear proximity using the nearest gene approach. Next, Pol III regulatory score for each mRNA gene was estimated based on the strengths of Pol III binding and their distance from the TSS of the corresponding mRNA. A nonparametric statistical test (Kolmogorov–Smirnov test) was used to compare regulatory scores for up-, downregulated, or non-changed genes on the basis of PRO-Seq or Pol II ChIP-Seq results before and after Pol III depletion.

### Gene Ontology and gene set enrichment analysis (GSEA)

Gene set enrichment analysis was performed using a pre-ranked gene list based on the difference (log2 fold change) detected in PRO-Seq between the untreated and Pol III degron samples by searching against the intronless genes (Fig. 3F) [97]. Normalized enrichment score (NES) and nominal *p* value were calculated from the result of 1000 permutations.

Gene Ontology analysis was completed using DAVID (v6.8) online tool with default settings to identify enriched terms in Pol III_degron elongation-affected mRNA genes (adjusted *p* value <0.05 and fold change ≤ −2) in mESCs (Fig. 3E) [98]. GO term categories were restricted to GOTERM_BP (Biological Process), GOTERM_MF (Molecular Function), GOTERM_CC (Cellular Component), and KEGG_PATHWAY.

### Metagene profiles, heatmaps, and volcano plots

For Fig. S1B, peak-centered heatmaps were calculated using deepTools computeMatrix with options “reference-point -a 5000 -b 5000”. The output matrix was plotted using the plotHeatmap for the region spanning −5 kb upstream to +5 kb downstream of TSS, ranked by descending ChIP-Seq read density of the corresponding factor.

For Fig. 4F and S2D, TSS-centered metagene profiles were generated using computeMatrix with options 'reference-point -b 5000 -a 5000' for the region spanning −5 kb upstream to +5 kb downstream of TSS followed by plotProfile, or centered at the TSS in a ±1-kb window for Figs. 4E, 5F, and S3A.

For Fig. 7A, the ratio of transcription rate was defined as divided Pol II ChIP-Seq signals by PRO-Seq signals, while PRO-Seq signals represent the nascent RNA synthesized during the nuclear run-on period, and Pol II ChIP-Seq measured the Pol II binding at the chromatin. Scaled metagene profiles for the ratio of Pol II ChIP-Seq over PRO-Seq or EU-seq signal were created using bamCompare with options “—binSize 10 --outFileFormat bigwig.” The computeMatrix with options “scale-regions -b 3000 -a 3000” and plotProfile commands of deepTools were used to produce aggregated metagene plots of the above ratio in the given genomic regions.

For Figs. 1C, E, 3B–D, and 4F, scaled metagene profiles of ChIP-Seq, ChAR-Seq, or PRO-Seq signals were produced using ngs.plot suite by dividing each gene into 100 equally sized bins, with a 3-kb flanking region on each side in bins of 50 bp. Read pairs sharing the same or opposite orientation as the gene strand were assigned as “sense” and “antisense,” respectively. Only the first mate of read pairs were extracted to make strand-specific metaplots, and the extreme 5% values were removed. The correct bam files were used to calculate read density across those bins and subsequently summarized for all protein-coding genes. For metagene plots besides the strand-specific ones mentioned above, default parameters of ngs.plot were used.

For Figs. 2C and 6C, differential analysis of ChIP-Seq binding intensities at indicated peak regions was defined as having an FDR-adjusted *p* value < 0.05 along with absolute foldchange > 2, and MA plots were produced by the DiffBind plotMA function.

For Fig. 5A, differential expression analysis was performed using DEseq2 with raw count data as input. Volcano plots of PRO-Seq were made based on the fold changes and *p* values derived from the Wald test on spike-in normalized and log2-transformed reads.

For Figs. 4D and 5E, the ATAC-Seq read counts for each sample at each tRNA region or mRNA promoter were obtained by featureCount. The resulting count matrix was analyzed with DEseq2 to produce differential tRNA or mRNA gene sets before and after individual RNA polymerase depletion. The analysis protocol matched that for PRO-Seq data (see “PRO-Seq and chromatin-associated RNA-Seq (ChAR-Seq) mapping and analysis” section), with equivalent thresholds for differential chromatin accessibility.

### Visualization of genome browser tracks

ChIP-Seq and ATAC-Seq signal tracks (bigwig format) were obtained for visualization on merged bam files with the command “bamCoverage --binSize 10 --normalizeUsing RPGC.”

For PRO-Seq and ChAR-Seq, Signal tracks were generated using the bamCoverage function in the deepTools package with options “--normalizeUsing RPKM.”

Figures illustrating these continuous signal tracks over selected genomic intervals were created in the Integrative Genomics Viewer (IGV) browser [99].

### ChIP-MS data analysis

The ChIP-MS analysis was done as described previously. Briefly, the gel was rehydrated three times in distilled water at room temperature for 10 min with gentle agitation. The protein bands were cut out and further cut off into ca 1 × 1 mm^2^ pieces, followed by reduction with 10 mM TCEP in 25 mM NH_4_HCO_3_ at 25 °C for 30 min, alkylation with 55 mM IAA in 25 mM NH_4_HCO_3_ solution at 25 °C in the dark for 30 min, and sequential digestion with trypsin at a concentration of 12.5 ng/mL at 37 °C overnight (1st digestion for 4 h and 2nd digestion for 12 h). Tryptic peptides were then extracted out from gel pieces by using 50% ACN/2.5% FA for three times, and the peptide solution was dried under vacuum. Dry peptides were purified by Pierce C18 Spin Tips (Thermo Fisher, USA).

For DDA-MS, Biognosys-11 iRT peptides (Biognosys, Schlieren, CH) were spiked into peptide samples at the final concentration of 10% prior to MS injection for RT calibration. Peptides were separated by Ultimate 3000 nanoLC-MS/MS system (Dionex LC-Packings, Thermo Fisher Scientific™, San Jose, USA) equipped with a 15 cm × 75 μm ID fused silica column packed with 1.9 μm 120 Å C18. After injection, 500 ng peptides were trapped at 6μL/min on a 20 mm × 75 μm ID trap column packed with 3 μm 100 Å C18 aqua in 0.1% formic acid, 2% ACN. Peptides were separated along a 60-min 3–28% linear LC gradient (buffer A: 2% ACN, 0.1% formic acid (Fisher Scientific), buffer B: 98% ACN, 0.1% formic acid) at the flowrate of 300 nL/min (108 min inject-to-inject in total). Eluting peptides were ionized at a potential of +1.8 kV into a Q-Exactive HF mass spectrometer (Thermo Fisher Scientific™, San Jose, USA). Intact masses were measured at resolution 60,000 (at m/z 200) in the orbitrap using an AGC target value of 3E6 charges and a maximum ion injection time of 80 ms. The top 20 peptide signals (charge-states higher than 2+ and lower than +6) were submitted to MS/MS in the HCD cell (1.6 amu isolation width, 27% normalized collision energy). MS/MS spectra were acquired at resolution 30,000 (at m/z 200) in the orbitrap using an AGC target value of 1E5 charges, and a maximum ion injection time of 100 ms. Dynamic exclusion was applied with a repeat count of 1 and an exclusion time of 30 s.

For DIA-MS, Biognosys-11 iRT peptides (Biognosys, Schlieren, CH) were spiked into peptide samples at the final concentration of 10% prior to MS injection for RT calibration. Peptides were separated at 300 nL/min in a 3–28% linear gradient (buffer A: 2% ACN, 0.1% FA, buffer B: 98% ACN, 0.1% FA) in 60 min (75 min inject-to-inject in total) for all samples. Eluting peptides were ionized at a potential of +1.8 kV into a Q-Exactive HF mass spectrometer (Thermo Fisher Scientific, San Jose, USA). A full MS scan was acquired analyzing 390–1010 m/z at resolution 60,000 (at m/z 200) in the orbitrap using an AGC target value of 3E6 charges and maximum IT 80 ms. After the MS scan, 24 MS/MS scans were acquired, each with a 30,000 resolution at m/z 200, AGC target 1E6 charges, and normalized collision energy was 27%, with the default charge state set to 2 and maximum IT set to auto. The cycle of 24 MS/MS scans (center of isolation window) with three kinds of wide isolation window are as follows (m/z): 410, 430, 450, 470, 490, 510, 530, 550, 570, 590, 610, 630, 650, 670, 690, 710, 730, 750, 770, 790, 820, 860, 910, 970.

To analyze DIA data, a DDA library was built by Spectronaut (version: 13.5.190902.43655). The library building was performed according to the standard workflow in Spectronaut (Manual for Spectronaut, available on the Biognosis website). Data was searched against the Swissprot Mouse database September 2018. The differential proteins of Cano MS were identified with the peptide-spectrum match (PSM) number, where all captured protein’s PSM were normalized to that of POLR2A in the same condition respectively as it was an immunoprecipitation target. After that, PSM of Pol III +IAA 1 h divided by untreated condition were calculated and ratio less than 0.8 was defined as decreased while greater than 1.2 was defined as increased protein. The differential proteins of DIA-MS were calculated by DEseq2 with protein abundance number with two biological replicates and cutoff with 1.2 fold change; protein abundance number is calculated by log2 of sum of top3 unique peptide numbers. Proteins localized outside of the nucleus were removed according to Uniprot annotation, and both of MS data were filtered where IgG IP or Input signal greater than samples were removed.

### Statistical analysis

Chromatin-associated RNA-Seq, ChIP-Seq, ChIP-MS, ChIP-qPCR, and RT-PCR and two biological replicates were conducted. *P* values and choice of statistical tests are reported in the figure legends, with the resulting numbers of observations indicated in the figure panels. Almost all the described data processing and analyzing steps (statistical tests, clustering, plotting, and so on) were performed in Python (v3.7.4) (www.python.org), the statistical computing environment R (v4.0.2) (www.r-project.org), and Microsoft Excel. Custom code used in this study is available upon request.

## Supplementary Information

Additional file 1: Supplementary Figures S1-S4.

Additional file 2: Table S1. Accession numbers of datasets used in this study.

Additional file 3: Table S2. Pol I/Pol II/Pol III ChIP-MS.

Additional file 4: Table S3. Next-generation sequencing mapping statistics.

Additional file 5: Table S4. ChIP-Seq analysis of differential Pol II peaks after depletion of Pol III.

Additional file 6: Table S5. PRO-Seq and Pol II ChIP-Seq defined elongation-affected and unaffected genes upon Pol III depletion.

Additional file 7: Table S6. ATAC-Seq analyses of differential mRNA chromatin accessibility after depletion of Pol I, II and III.

Additional file 8: Table S7. PRO-Seq analysis of differential tRNA expression after depletion of Pol I, II and III.

Additional file 9: Table S8. ATAC-Seq analyses of differential tRNA chromatin accessibility after depletion of Pol I, II and III.

Additional file 10: Table S9. Pol III_degron_anti-Pol II DDA ChIP-MS.

Additional file 11: Table S10. Pol III_degron_anti-Pol II DIA ChIP-MS.

Additional file 12: Table S11. Oligos used in this study.

Additional file 13. Uncropped images for western blot.

Additional file 14. Review history.

## Data Availability

All the sequencing datasets generated in this study, including the raw and processed ChIP-Seq, ATAC-Seq, PRO-Seq, ChAR-Seq, EU-Seq, and RNA-Seq data, have been deposited in GEO [100]. Raw sequencing data can be found in the GEO database: GSE181701. Raw sequencing data of native elongating transcript sequencing (NET-Seq) for a previously published FACT knockdown experiment in Fig. 6H can be found in the GEO database: GSE90906 [101].
