# Supplementary material for: Cross-regulome profiling of RNA polymerases highlights the regulatory role of polymerase III on mRNA transcription by maintaining local chromatin architecture
Source: Genome Biol. 2022 Nov 28;23:246. doi: 10.1186/s13059-022-02812-w (PMC9703767; doi:10.1186/s13059-022-02812-w)
Supplement: Supplementary file 1 — Additional file 1: Supplementary Figures S1-S4. [file 13059_2022_2812_MOESM1_ESM.docx]

**Supplementary Figures for “Cross-regulome profiling of RNA polymerases highlights the regulatory role of polymerase III on mRNA transcription by maintaining local chromatin architecture”**





**Figure S1. Pol I, Pol II, and Pol III mostly occupy specific genomic regions but share many transcriptional regulators.**

1. Genome browser ChIP-Seq tracks of Pol I, Pol II and Pol III at the 39,840,997–39,850,829 region on chromosome 17, the 105,152,518–105,310,247 region on chromosome 11 and the 21,158,677–21,164,058 region on chromosome 13 immunoprecipitated with their corresponding endogenous antibody. The input is shown in the bottom panel, and the y-axis shows the normalized read density in reads per genome coverage (RPGC).
2. Quality control of ChIP-Seq datasets, and Pol I, II, and III ChIP-Seq was performed in mESCs in two replicates. An individual cross-correlation plot for the respective ChIP-Seq sample is shown with NSC and RSC values indicated below, suggesting that our data are of high quality. NSC represents the normalized strand coefficient, while RSC represents the relative strand coefficient.
3. The coverage histogram depicts the sequencing depths for each RNA Pol of two independent replicates.
4. Heatmap showing the relative enrichment of genomic features for Pol I, Pol II and Pol III ChIP-Seq.
5. Heatmaps showing the enrichment of Pol I, Pol II, and Pol III at their specific and common peaks as previously defined in mESCs, with the corresponding input signal included as a control. The peaks were organized into seven groups based on the combinatorial binding of the three RNA polymerases, with the numbers indicated on the left side. For example, G1–G3 represent loci bound only by Pol I, Pol II or Pol III, G4 represents loci bound only by Pol I and Pol II, and G7 represents loci bound by all three polymerases. The rows in the heatmaps show genes ranked according to decreasing read density within a ± 5 kb region centered on the peak summits under the untreated condition. Each row of a heatmap represents an individual region of interest with the ChIP-Seq signal profile, displayed in normalized reads per million in a ± 5 kb region centered on that region of interest. The color bar at the bottom indicates the range of ChIP-Seq signals per 50 bp bin pooled from two independent biological replicates. The average signal levels around the indicated regions of those seven groups were calculated and presented as metaplots and box plots shown in the right panel.
6. Pie charts showing the genomic distribution of the indicated groups are shown in Fig. S1E.
7. Heatmap displaying the Pearson correlation coefficients of Pol I, Pol II, and Pol III ChIP-seq signals with various genomic and epigenetic features at promoters or gene body regions in wild-type mESCs with publicly available datasets.





**Figure S2. Cross-regulation between Pol II and Pol III mediated by chromatin structure alterations.**

1. Mass spectrometry of anti-GFP immunoprecipitates obtained from the crosslinked chromatin fractions of Pol I_degron, Pol II_degron, Pol III_degron and wild-type mESCs and a heatmap showing the log2(fold change) values of unique peptides with respect to input normalized by the z score. Transcription regulators are shown.
2. The same analysis described in Fig. S2A and subunit compositions of Pol I, Pol II and Pol III are shown.
3. Western blot analysis of RPAC1 protein levels in RPAC1_degron mESCs at different time points after IAA treatment. TIR1-HA protein levels were also examined to indicate efficient induction by doxycycline. b-Actin served as the loading control.
4. Upper panel, heatmap of the normalized Pol III degron PRO-seq and EU-seq signals centered at the Pol III peaks (n = 1845), showing a reduction in Pol III binding peaks upon its depletion. Heatmaps are ordered by descending Pol III ChIP-seq signal intensity; bottom panel, genome browser track of PRO-seq and EU-seq under untreated and after 1 hr of IAA treatment conditions in several identified Pol III peaks in Pol III degron cells. The y- axis shows the normalized read density in reads per genome coverage (RPGC).
5. Schematic workflow for Pol III peak classification and Pol II-affected gene grouping.
6. Sankey plots showing the Pol III peaks (C, E, F, and G as defined in the Venn Diagram of Fig. S2E) classified by loop, distance, enhancers and regulatory elements. Loop: Pol III peaks defined with or without overlapping HiChIP loops. Distance: Pol III peaks identified by nearby (distance of 10 kb) annotated genes. Enhancers: Pol III peaks identified with or without overlapping enhancers. Elements: Pol III peaks identified with or without overlapping regulatory elements, such as SINEs, tRNAs or ncRNAs.
7. ATAC-Seq metaplots of open chromatin over the promoter regions of genebody-up mRNAs (N = 773, blue), genebody-unaffected mRNAs (N = 3290, green), and unchanged mRNAs (N= 4782, gray) (left) upon Pol III depletion.
8. Western blot analysis of anti-Pol II immunoprecipitates obtained from native chromatin fractions of untreated Pol III_degron cells and Pol III_degron cells after 1 hr of IAA treatment revealed no significant change in Pol II subunit composition after Pol III depletion. The RPB-X factors examined here are Pol II subunits.


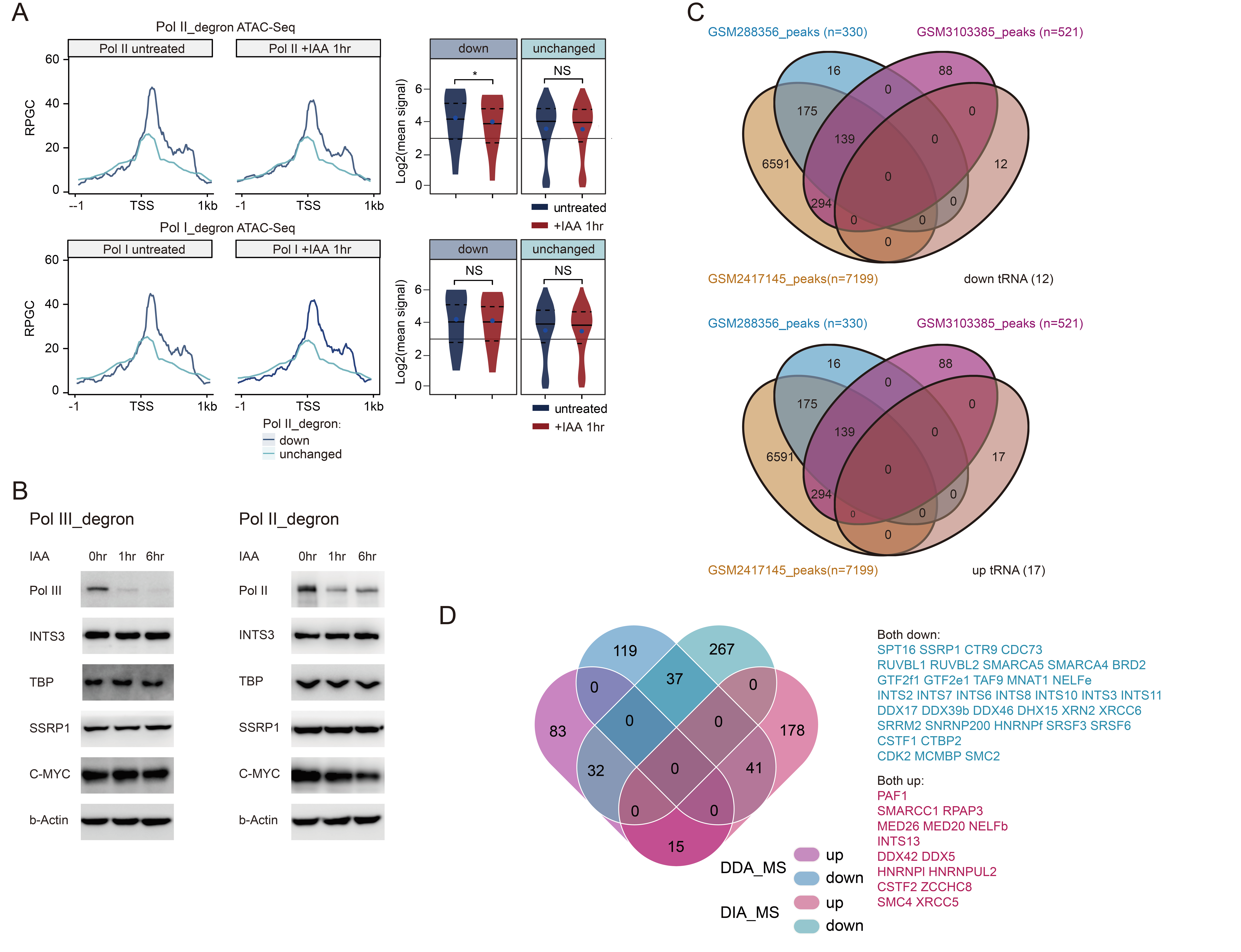


**Figure S3. Pol III depletion decreases the chromatin binding for the FACT complex.**

1. The ATAC-Seq metaplots of open chromatin and the half-violin plots shown are the same as those described in Fig. S2G but are based on the downregulated (N = 12, blue) or unchanged (N = 244, green) tRNA genes according to their transcription levels determined by PRO-Seq upon Pol II depletion.
2. Western blot analysis of INTS3, TBP, SSRP1 and C-MYC protein levels in whole-cell lysates at different time points after IAA treatment in Pol II_degron and Pol III_degron mESCs. b-Actin served as the loading control.
3. Four-way Venn diagram comparing the overlap of three independent C-MYC binding peaks from publicly available ChIP-Seq data in mESCs and downregulated (left) and upregulated (right) tRNAs from Pol II degron PRO-Seq.
4. Overlap analyses of Pol II-interacting proteins that are upregulated and downregulated upon Pol III depletion in both canonical DDA and DIA MS datasets. Thirty-seven proteins showed decreased interaction with Pol II upon Pol III depletion, and 15 proteins showed increased interaction with Pol II in both MS experiments. See the detailed procedure in the Supplementary Methods section.





**Figure S4. Pol III depletion decreases the Pol II transcription rate.**

1. Heatmaps and metaplots of TBP, INTS3, and SSRP1 ChIP-Seq in Pol III_degron cells under untreated (left) and after 1 hr of IAA treatment (right) conditions centered at the TSS of Pol III degron genebody-up genes ranked by decreasing occupancy.
2. Genome browser of ATAC-Seq and ChIP-Seq tracks at the same region as Fig. 3G, and the y-axis shows the normalized read density in reads per genome coverage (RPGC).
3. Genome browser track at the 103,104,177-103,384,370 region on chromosome 14 for Pol II ChIP-Seq signals in Pol III_degron and RPAC1_degron cells and SSRP1 ChIP-Seq signals in Pol III_degron cells that were untreated or treated with IAA for 1 hr. PRO-Seq and ATAC-Seq in Pol III_degron cells that were untreated or treated with IAA for 1 hr and Pol III ChIP-Seq in wild-type mESCs are shown in the same region. Only sense strand signals of PRO-Seq and ChAR-Seq are presented, and all tracks are flipped horizontally.
4. Bar graphs showing relative ChIP enrichment normalized to input (5%) at the locus indicated in the upper panel. Each sample was analyzed with two technical replicates per biological replicate and two biological replicates in total. Statistical significance was evaluated by Student’s t test (**: <0.01, *: <0.05).
5. Reads per genome coverage (RPGC)-normalized count quantification of ATAC-seq and SSRP1 ChIP-Seq at the promoter region and Pol II ChIP-Seq and PRO-Seq in the gene body region upon Pol III or RPAC1 depletion. Statistical significance was evaluated by Student’s t test (**: <0.01, *: <0.05).
6. Metagene profiles of EU-Seq signals over the genebody-up (blue) and genebody-unaffected (green) genes in 1 hr of IAA-treated versus untreated RPC1_degron (upper panel) and RPAC1_degron cells (lower panel), with violin plots shown on the right measuring the changes in ChIP-seq signals between IAA-treated (blue) and untreated (red) samples for genebody-up and -unaffected gene sets. P values were calculated using a Wilcoxon rank-sum test. The y-axis displays the normalized read density in reads per genome coverage (RPGC).
