# Supplementary figures and images for: Cross-regulome profiling of RNA polymerases highlights the regulatory role of polymerase III on mRNA transcription by maintaining local chromatin architecture
Source: Genome Biol. 2022 Nov 28;23:246. doi: 10.1186/s13059-022-02812-w (PMC9703767; doi:10.1186/s13059-022-02812-w)

Figure 1A

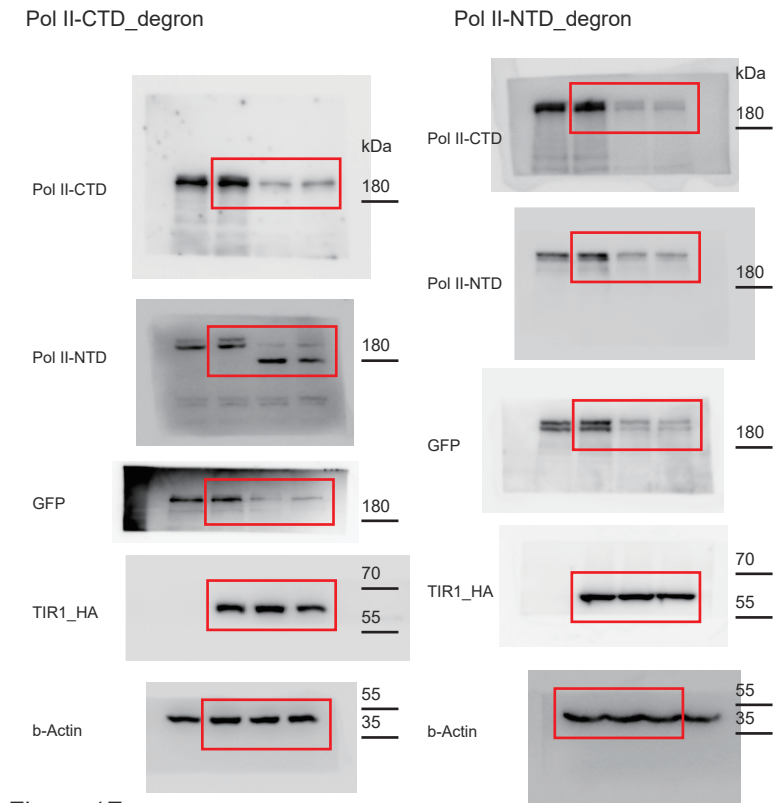

Figure 6B

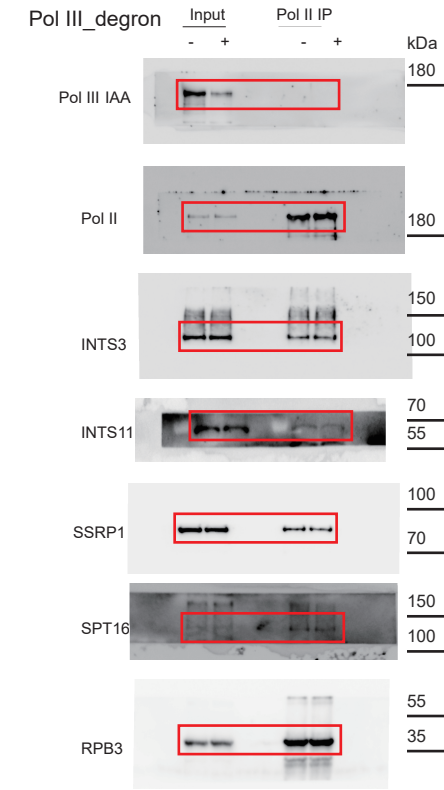

Figure 1F

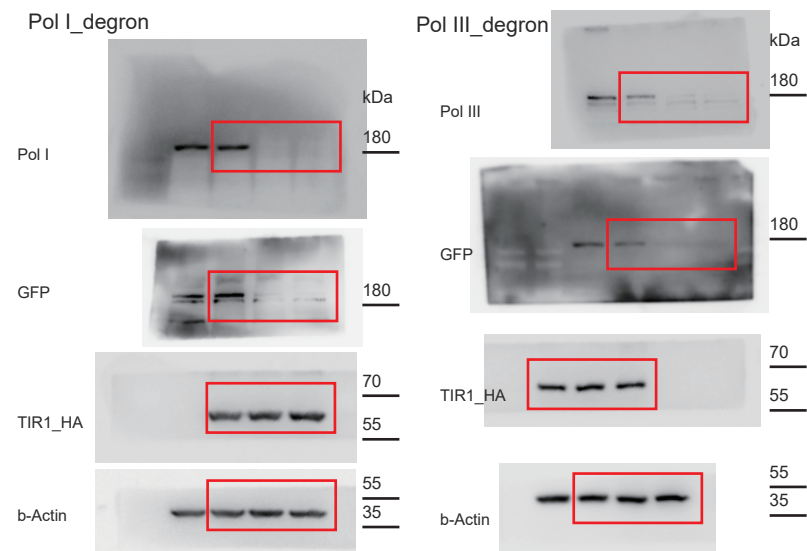

Figure S2C

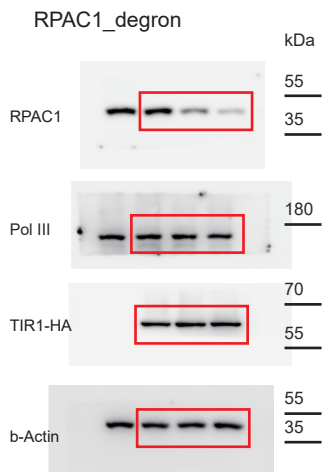

Figure S3B

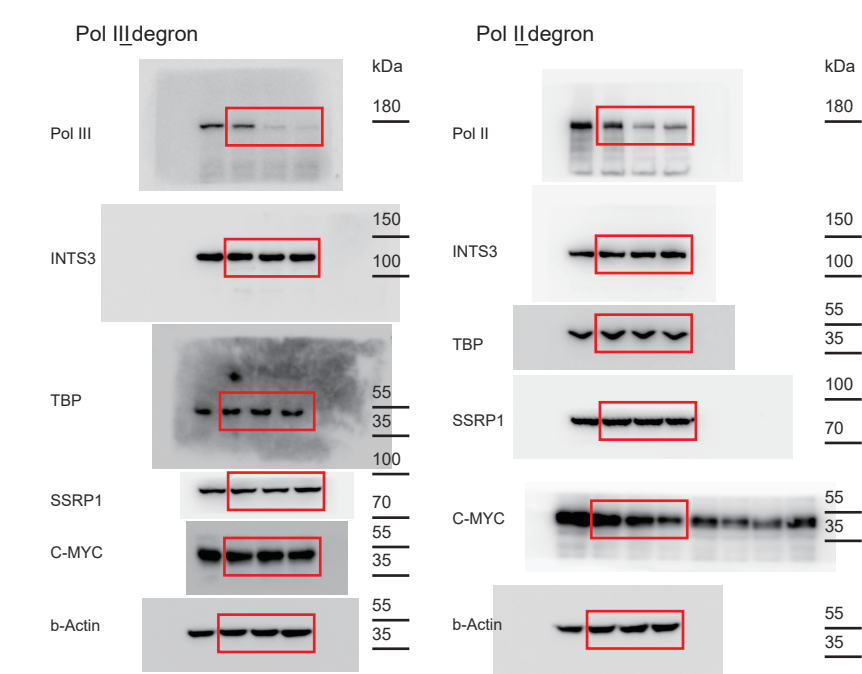

Figure S2H

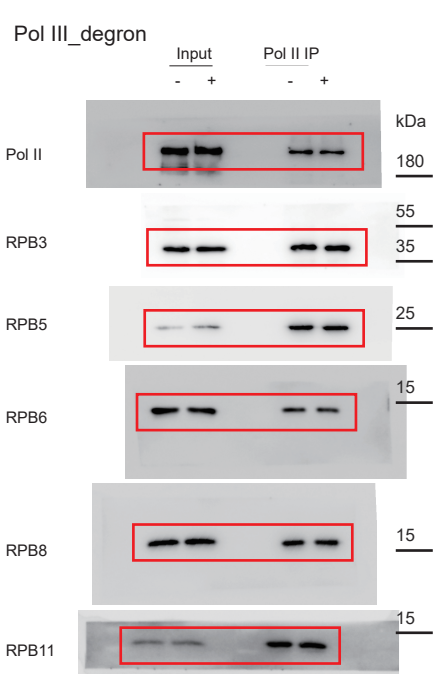

Supplement: Supplementary file 13 — Additional file 13. Uncropped images for western blot. [file 13059_2022_2812_MOESM13_ESM.pdf]
