# Supplementary material for: Cross-regulome profiling of RNA polymerases highlights the regulatory role of polymerase III on mRNA transcription by maintaining local chromatin architecture
Source: Genome Biol. 2022 Nov 28;23:246. doi: 10.1186/s13059-022-02812-w (PMC9703767; doi:10.1186/s13059-022-02812-w)
Supplement: Supplementary file 14 — Additional file 14. Review history. [file 13059_2022_2812_MOESM14_ESM.docx]

Review History

**First round of review**

**Reviewer 1**

**Were you able to assess all statistics in the manuscript, including the appropriateness of statistical tests used?**

No, I do not feel adequately qualified to assess the statistics.

**Comments to author:**

In this study Jiang and coauthors assess whether there is cross-regulation between the three types of RNA polymerases by analyzing transcription by each Pol upon acute depletion of the others. They observe that Pol III depletion has considerable effect on Pol II transcription and propose that Pol III binding near the promoter of a subset of Pol II-dependent genes promotes the recruitment of the FACT complex, which in turns facilitates Pol II progression. The authors generated an enormous amount of data and certainly made some interesting observations. However, their final model remains supported by correlations (some stronger than others) and some key results, for instance those related to the decrease in FACT recruitment upon Pol III depletion, which is one of the main messages of the paper, are not very solid.

Major comments:

-Regarding the Pol III peaks they detect by ChIP: it is known since long that the ChIP technique provides a good amount of "false positives" or "phantom peaks" that often coincide with gene promoters (https://www.nature.com/articles/s41592-019-0365-3). It does not seem that the authors of this study have performed any extra validation to be confident that the non-annotated Pol III peaks they detect do reflect true Pol III binding. A good validation could be to perform ChIP experiments upon Pol III depletion to verify that the peaks decrease or disappear. At least ChIP-qPCR should be done on several cases because this point is very important given that the whole model is based on the idea that Pol III binding near promoters alter locally the chromatin structure.

Related to this point, in the discussion the authors propose that the identified Pol III peaks correspond to transcribing Pol III. If that were the case they should be able to detect RNAs mapping at those sites, either in RNA-seq experimets or at least in Chr-RNAseq datasets if the produced RNAs are rapidly degraded.

-All along the manuscript the authors talk about cross-regulation, but for regulation to be effective the levels of the produced transcripts should significantly change upon Pol III depletion. The authors should show RNA-seq data (that they generated) indicating that the Pol II genes that are presumably cross-regulated by Pol III present not only a different Pol II pattern, but also different expression levels.

-Line 118: "Pol I, Pol II and Pol III share many transcriptional regulators". I do not think they can conclude this from their ChIP-MS experiments (figure S1). Pol I, II and III transcription units can be found in the vicinity of genes transcribed by the other polymerases, so they might pick up common regulators simply because of that. The formaldehyde treatment provokes the formation of big protein-nucleic acid aggregates, so it is likely that by immunoprecipitating one type of polymerase they pick up neighbor chromatin regions transcribed by other type of polymerase. Indeed, there is some contamination of each sample with the other polymerases.

-Figure 2A: the changes they observe are very minor and rather look like a subtle redistribution of the signal. The results could be more convincing if the authors showed the signal in various biological replicates so that readers can appreciate whether the differences between conditions are reproducible and not found between biological replicates of the same condition.

Regarding the proposed decrease in FACT recruitment upon Pol III depletion:

- Figure 6A: in the legend there is no information about the number of replicates and statistical analyses they performed to conclude that the differences they observe between the untreated and IAA-treated cells are significant. According to the way they describe the experiment it does not seem that it can actually provide quantitative information about the recruitment of FACT and Pol II-transcribed regions.

- Figure 6B: the quantitative power of western blots is very limited and the decrease in SSRP1 and SPT16 is not convincing.

On the contrary, the effect they observe on SSRP1 recruitment by ChIP is rather massive, to the point that one could ask how it is possible that this effect is linked to the decreased binding of Pol III to a relatively few sites.

The conclusions would probably be more convincing if the authors showed SSRP1 ChIP-seq data for several individual examples (only Nipbl was shown and it is not clear whether Pol III binds close to its promoter). Showing the fold change (FC) or the log2 FC between treated and untreated conditions can help readers to appreciate whether the change in the signal is a real increase/decrease or just a slightly different distribution.

Also related to FACT recruitment, upon Pol III depletion at genebody-up genes the authors observe by ChIP decreased binding of Pol II at the promoter with concomitant decrease in SSRP1 ChIP signal and higher nucleosome occupancy. They conclude that FACT recruitment is compromised because of the absence of Pol III binding nearby and that this is the cause of the different Pol II pattern. However, it could very well be that it is the decrease in Pol II binding to the promoter or a decrease in the initiation rate that provokes a reduction in FACT recruitment and higher nucleosome persistence. Indeed, in a recent article in the yeast model, it has been proposed that FACT is recruited to the +1 nucleosome once it is partially unwrapped by Pol II (PMID: 34380014). If that were the case, their entire model would not be correct and it would remain completely unclear why Pol III depletion leads to different Pol II signals at as subset of genes.

-Writing needs more than polishing. There are a number of sentences that are grammatically incorrect (e.g. lacking a verb) and paragraphs that are not clear. Some examples:

- Legend of figure 4: "the unchanged genes are genes that did not be affected by Pol III depletion at all. Detailes see Methods)"

- Lines 185-187: "Further overlap analyses showed that the peaks changed in the Pol II ChIP-Seq data after Pol III depletion had the most overlap with the Pol III ChIP-Seq peaks in both the ChIP-Seq and PRO-Seq datasets". I do not understand what this means.

- Lines 207-210: " The genebody-up genes were identified by overlapping genes with

upregulated signals after Pol III depletion identified by both Pol II ChIP-Seq and

PRO-Seq of gene bodies, and genebody-unaffected genes were identified by

overlapping the genes with unchanged signals under the same conditions". This paragraph is not very clear. I suggest to reformulate as follows (if I understood well): genes with increased Pol II ChIP-seq and PRO-Seq signal at the gene body were designated genebody-up genes... etc

- Lines 263-265 : " As retrotransposon elements SINE encoding non-coding RNAs transcribed by Pol III, and that were previously proposed to link to Pol II through chromatin looping."

-Related to writing, most legends are not clear, so it is quite difficult to understand the data presented. Also, there are some mistakes. For instance, in the figure 4 legend they say mRNAs while they are talking about genes.

Other comments/concerns:

- Line 213: Pausing index. I guess the authors mean promoter-proximal pausing index.

Related to this, their conclusion that "depletion of Pol III leads to a widespread decrease in Pol II initiation at promoter regions, while increases Pol II signals at gene bodies for a subset of genes" is weird in the light of their results. Considering that they detect either increased signal or unchanged signal at gene bodies and that the Pol II signal in gene bodies should in principle derive from initiation events, I think the only thing they can propose at this stage is that promoter-proximal pausing is decreased upon Pol III depletion.

-Line 220: they talk about "PRO-Seq signals at the promoter" but PRO-Seq does not detect Pol II at the promoter. Since it sequences the nascent RNA, it can only detect polymerases that have initiated transcription.

-The authors often confound correlations with causal connections. For instance, in lines 268-271 they say "We then annotated the Pol III binding sites that have regulatory roles on Pol II, and identified 121 (15.7%), 68 (8.9%), and 34 (4.4%) genebody-up genes (N=773) that could be explained by transcriptional interference, chromatin looping, and ncRNA models". It seems to me that they just hypothesize that these genes are regulated by Pol III by the indicated mechanisms based of the position of Pol III peaks relative to each genebody-up gene. They should clarify this in the text.

-Figure 6E: I'm not sure how to interpret these data, the authors could explain a bit more the rationale of this analysis and indicate more clearly what the y axis represents.

**Reviewer 2**

**Were you able to assess all statistics in the manuscript, including the appropriateness of statistical tests used?**

Yes, and I have assessed the statistics in my report.

**Comments to author:**

In this paper the authors use the degron system to tag and rapidly degrade the primary subunit of RNA polymerase I, II, and III. The authors show clear evidence that their degron tag works to degrade the appropriate RNA polymerase submit. They use these cell lines, in conjunction with ChIP-seq, PRO-seq, and Hi-Chip data to explore how perturbation of RNA polymerase I, II, and III affects each of the other RNA polymerases. The authors generally find that Pol III has an impact on Pol II transcription in a variety of locations across the genome, including some previously identified loci. The authors go on to argue that Pol III depletion affects elongation rate through FACT deposition in Pol II. This paper is interesting, and in general it is well-written. Results are generally well-supported by the data. My main comment is that the writing is dense, and some of the results are difficult to follow. I would encourage the authors to go through the manuscript with an eye toward additional clarity of the presentation.

Additional comments:

Could ChIP-seq sites in which all three RNA Pols overlap be driven by mapping artifacts? Have ENCODE blacklist regions (and other similar sites) been removed prior to analysis?

Line 287 - the topic starts with decreased nucleosome positioning, but no data is shown (only general accessibility). Are the authors talking about accessibility (i.e., the amount of signal)? Or nucleosome positioning?

Pp. 6: The switch in language from "clusters" to "groups" is confusing. Consider renaming "group x" to "cluster x".

**Authors Response**

**Point-by-point responses to the reviewers’ comments:**

**Reviewer #1:**

In this study Jiang and coauthors assess whether there is cross-regulation between the three types of RNA polymerases by analyzing transcription by each Pol upon acute depletion of the others. They observe that Pol III depletion has considerable effect on Pol II transcription and propose that Pol III binding near the promoter of a subset of Pol II-dependent genes promotes the recruitment of the FACT complex, which in turns facilitates Pol II progression. The authors generated an enormous amount of data and certainly made some interesting observations. However, their final model remains supported by correlations (some stronger than others) and some key results, for instance those related to the decrease in FACT recruitment upon Pol III depletion, which is one of the main messages of the paper, are not very solid.

***Response:*** *We agree with the reviewer that our results are correlated with the final model and appreciate the constructive suggestions to further strengthen our conclusion. We have performed all the experiments and analyses suggested by the reviewer and regret that we confused the reviewer due to a lack of clarity in the original manuscript. We proposed a model in the original manuscript, and we agree that multiple models should help to explain the cross-regulation between Pol III and Pol II. In the revised manuscript, we have further toned down our statement to emphasize the interesting observations reported in our study, concluded that our results are correlated with the FACT-Pol II axis model, and discussed that other models could also explain our results.*

Major comments:

-Regarding the Pol III peaks they detect by ChIP: it is known since long that the ChIP technique provides a good amount of "false positives" or "phantom peaks" that often coincide with gene promoters (https://www.nature.com/articles/s41592-019-0365-3). It does not seem that the authors of this study have performed any extra validation to be confident that the non-annotated Pol III peaks they detect do reflect true Pol III binding. A good validation could be to perform ChIP experiments upon Pol III depletion to verify that the peaks decrease or disappear. At least ChIP-qPCR should be done on several cases because this point is very important given that the whole model is based on the idea that Pol III binding near promoters alter locally the chromatin structure. Related to this point, in the discussion the authors propose that the identified Pol III peaks correspond to transcribing Pol III. If that were the case they should be able to detect RNAs mapping at those sites, either in RNA-seq experimets or at least in Chr-RNAseq datasets if the produced RNAs are rapidly degraded.

***Response:*** *We agree with the reviewer that it is crucial to validate the Pol III peaks detected by ChIP. The Pol III peaks have been validated in three different aspects. 1) We previously showed that Pol III binding significantly decreased at the identified Pol III peaks after Pol III depletion [1]. 2) As suggested by the reviewer, we further validated the Pol III peaks with Pol III ChIP–qPCR upon Pol III depletion at the Bub1, Nipbl and Mycbp2 regions. The results showed that Pol III binding significantly decreased upon Pol III depletion in a time-dependent manner (see below, Figure R5). 3) As our RNA-Seq captured poly-A RNAs that would be absent in Pol III transcripts, we then quantified the PRO-seq and ChAR-seq signals at the Pol III peaks and showed that these RNAs decreased after 1 hr of depletion of Pol III (Figure R1). The reason that the RNAs decreased but were not eliminated may be that the adjacent Pol II transcribed RNAs interfered with the quantification.*

*In the revision, we added a heatmap to illustrate PRO-seq and ChAR-seq signal changes at identified Pol III peaks upon Pol III depletion (New Figure S2D). Both of them showed a significant decrease upon Pol III depletion. In addition, we verified Pol III binding with ChIP–qPCR in the presented examples, which showed efficient Pol III depletion upon IAA treatment at different time points (New Figure 6F).*

**Figure R1****.** Heatmap of the normalized Pol III degron PRO-seq and ChAR-seq signals centered at the Pol III peaks (n = 1845), showing the marked reduction in Pol III binding upon its depletion. Heatmaps are ordered by descending Pol III ChIP-Seq signal intensity. The PRO-seq signals appeared at most of the Pol III binding sites, suggesting that these regions were actively transcribed. The ChAR-seq signals did not appear for all Pol III binding sites, indicating that subsets of Pol III transcribed RNAs had short half-lives. The changes in ChAR-seq were relatively less than those in PRO-seq because chromatin-associated RNA sequencing would also capture some chromatin-associated noncoding RNAs in addition to nascent RNAs.

***Reference:***

*1.	Jiang Y, Huang J, Lun K, Li B, Zheng H, Li Y, Zhou R, Duan W, Wang C, Feng Y, et al: Genome-wide analyses of chromatin interactions after the loss of Pol I, Pol II, and Pol III. Genome Biol 2020, 21:158.*

-All along the manuscript the authors talk about cross-regulation, but for regulation to be effective the levels of the produced transcripts should significantly change upon Pol III depletion. The authors should show RNA-seq data (that they generated) indicating that the Pol II genes that are presumably cross-regulated by Pol III present not only a different Pol II pattern, but also different expression levels.

***Response:*** *Pol III depletion slows down the Pol II transcription rate, as we showed, and the produced transcripts gradually decrease with time upon Pol III depletion. We performed polyA RNA-seq after Pol III depletion for 24 hr, 48 hr and 96 hr. The heatmap showed that the genebody -up genes upon Pol III depletion did show a gradual decrease in mRNA expression levels by time-series RNA-seq (Figure R2). A subset of genes did not decrease, perhaps due to the secondary effects after long-term depletion of Pol III. That is also why we used nascent RNA sequencing with acute depletion in the original manuscript.*

*In the revision, we added a heatmap to illustrate the mature transcript levels of the genebody -up genes upon Pol III depletion (n=773) from time-series RNA-seq data (Pol III depletion of 0, 24, 48 and 96 hr with two biological replicates) (New Figure 7E), which showed a gradual decrease in static mRNA expression levels.*

**Figure R2**. Heatmap representing the gene expression levels of the genebody -up genes upon Pol III depletion (n=773) from time-series RNA-seq data (Pol III depletion for 0, 24, 48 and 96 hr with two biological replicates). Each row represents the z score transformed log2 (FPKM+1) values for one gene across different time points (green, low expression; red, high expression).

-Line 118: "Pol I, Pol II and Pol III share many transcriptional regulators". I do not think they can conclude this from their ChIP-MS experiments (figure S1). Pol I, II and III transcription units can be found in the vicinity of genes transcribed by the other polymerases, so they might pick up common regulators simply because of that. The formaldehyde treatment provokes the formation of big protein-nucleic acid aggregates, so it is likely that by immunoprecipitating one type of polymerase they pick up neighbor chromatin regions transcribed by other type of polymerase. Indeed, there is some contamination of each sample with the other polymerases.

***Response:*** *We agree with the reviewer that it has been shown that* *RNA polymerases could be found in close association with chromatin [2-5], so ChIP-MS experiments with formaldehyde crosslinking may create false positive interactions.* *Close chromatin binding of different RNA polymerases in some loci may create opportunities for biologically meaningful cross-regulatory effects, as reported previously [6-10], where some transcriptional regulators coordinate the transcription of different RNA polymerases. The goal of the analyses in Figure S1 was to provide candidate regulators as mentioned above. We regret causing confusion due to the incorrect statement and thank the reviewer for the careful review of our manuscript. For clarity, we deleted the original sentence that the reviewer pointed out and added the new sentence “**Pol I, Pol II and Pol III mostly occupy specific genomic regions, but close associations exist at specific loci”. The limitations of ChIP-MS data for transcriptional regulators have also been discussed in the revised manuscript.*

***References:***

*2.	Barski A, Chepelev I, Liko D, Cuddapah S, Fleming AB, Birch J, Cui K, White RJ, Zhao K: Pol II and its associated epigenetic marks are present at Pol III-transcribed noncoding RNA genes. Nat Struct Mol Biol 2010, 17:629-634.*

*3.	Moqtaderi Z, Wang J, Raha D, White RJ, Snyder M, Weng Z, Struhl K: Genomic binding profiles of functionally distinct RNA polymerase III transcription complexes in human cells. Nat Struct Mol Biol 2010, 17:635-640.*

*4.	Oler AJ, Alla RK, Roberts DN, Wong A, Hollenhorst PC, Chandler KJ, Cassiday PA, Nelson CA, Hagedorn CH, Graves BJ, Cairns BR: Human RNA polymerase III transcriptomes and relationships to Pol II promoter chromatin and enhancer-binding factors. Nat Struct Mol Biol 2010, 17:620-628.*

*5.	Raha D, Wang Z, Moqtaderi Z, Wu L, Zhong G, Gerstein M, Struhl K, Snyder M: Close association of RNA polymerase II and many transcription factors with Pol III genes. Proc Natl Acad Sci U S A 2010, 107:3639-3644.*

*6.	James Faresse N, Canella D, Praz V, Michaud J, Romascano D, Hernandez N: Genomic study of RNA polymerase II and III SNAPc-bound promoters reveals a gene transcribed by both enzymes and a broad use of common activators. PLoS Genet 2012, 8:e1003028.*

*7.	Hoogstraten D, Nigg AL, Heath H, Mullenders LH, van Driel R, Hoeijmakers JH, Vermeulen W, Houtsmuller AB: Rapid switching of TFIIH between RNA polymerase I and II transcription and DNA repair in vivo. Mol Cell 2002, 10:1163-1174.*

*8.	Wlotzka W, Kudla G, Granneman S, Tollervey D: The nuclear RNA polymerase II surveillance system targets polymerase III transcripts. Embo j 2011, 30:1790-1803.*

*9.	Dergai O, Hernandez N: How to Recruit the Correct RNA Polymerase? Lessons from snRNA Genes. Trends Genet 2019, 35:457-469.*

*10.	Bhalla P, Vernekar DV, Gilquin B, Couté Y, Bhargava P: Interactome of the yeast RNA polymerase III transcription machinery constitutes several chromatin modifiers and regulators of the genes transcribed by RNA polymerase II. Gene 2019, 702:205-214.*

-Figure 2A: the changes they observe are very minor and rather look like a subtle redistribution of the signal. The results could be more convincing if the authors showed the signal in various biological replicates so that readers can appreciate whether the differences between conditions are reproducible and not found between biological replicates of the same condition.

***Response:*** *The cross-regulations shown in Figure 2A have been demonstrated previously in several independent studies: Pol II-mediated R-loops prevent Pol I transcription at large intergenic spacer (IGS) regions in the nucleolus, and transcriptional interference occurs between Pol II and its intragenic MIR unit transcribed by Pol III [11-13]. Our ChIP-Seq data validated these results independently in a different cell line and further supported these phenomena by ChIP–qPCR (Figure 2B), which also confirmed our system and conclusion. As the reviewer suggested, we showed two biological replicates, and the result is reproducible as indicated (Figure R3).*

**Figure R3**. Genome browser track of Pol I ChIP-Seq signals at rRNA cDNA units in Pol II_degron cells under untreated conditions and after 1 hr of IAA treatment to visualize the IGS regions (see Methods for details, top). Genome browser track of Pol II ChIP-Seq signals in Pol III_degron cells or Pol III ChIP-Seq signals in Pol II_degron cells in the 120,914,905–120,924,630 region on chromosome 7 under untreated conditions and after 1 hr of IAA treatment (bottom). The y-axis shows the normalized read density in reads per genome coverage (RPGC). Note that the intergenic spacer (IGS) region is magnified at the bottom to show the details. Two biological replicates are shown.

***References:***

*11.	Abraham KJ, Khosraviani N, Chan JNY, Gorthi A, Samman A, Zhao DY, Wang M, Bokros M, Vidya E, Ostrowski LA, et al: Nucleolar RNA polymerase II drives ribosome biogenesis. Nature 2020, 585:298-302.*

*12.	Yeganeh M, Praz V, Cousin P, Hernandez N: Transcriptional interference by RNA polymerase III affects expression of the Polr3e gene. Genes Dev 2017, 31:413-421.*

*13.	Gerber A, Ito K, Chu CS, Roeder RG: Gene-Specific Control of tRNA Expression by RNA Polymerase II. Mol Cell 2020, 78:765-778.e767.*

Regarding the proposed decrease in FACT recruitment upon Pol III depletion:

-Figure 6A: in the legend there is no information about the number of replicates and statistical analyses they performed to conclude that the differences they observe between the untreated and IAA-treated cells are significant. According to the way they describe the experiment it does not seem that it can actually provide quantitative information about the recruitment of FACT and Pol II-transcribed regions.

***Response:*** *We thank the reviewer for the comments. The ChIP-MS results in the original Figure 6A were plotted with peptide-spectrum match (PSM) values identified by canonical data-dependent acquisition (DDA)-based mass spectrometry and were not quantitative, and we used these results as a screening resource for candidate regulators. That is why there were no statistical analyses in the original manuscript. For a more solid conclusion, in the revised manuscript, we report data-independent acquisition (DIA) mass spectrometry for Pol II ChIP samples upon Pol III depletion with two biological replicates. DIA is a relative quantitative mass spectrometry method that acquires survey scan (MS1) data and all detected precursor ions within MS1 fragment (MS2) data without bias to precursor ion selection, offering better reproducibility and quantitative sensitivity than the canonical DDA-based MS method [14, 15]. After differential analysis with both MS datasets, we identified increased and decreased protein interactions with Pol II upon Pol III depletion. We compared the differential proteins of DIA MS with those of canonical DDA MS, and these two experiments showed overlap with 37 downregulated and 15 upregulated proteins, which included transcriptional regulators such as FACT, Integrator and some other factors (Figure R4). The overlapping differential proteins were picked up and clustered with Cytoscape to replace the old version presentation in Figure 6A (Figure R5).*

*In the revision, we replaced the ChIP-MS result in Figure 6A with the canonical DDA and DIA MS overlapping differential proteins. In addition, a Venn diagram was used as a supplementary new Figure S3B. The detailed procedure and statistical analyses are described in the methods section of the revised manuscript.*

**Figure R4**. Overlap analyses of upregulated and downregulated proteins interacting with Pol II upon Pol III depletion identified in both canonical DDA and DIA MS datasets. Thirty-seven proteins showed decreased interaction with Pol II upon Pol III depletion, and 15 proteins showed increased interaction with Pol II in both MS experiments. See the detailed procedure in the supplementary Methods section.

**Figure R5**. Experimental setup for mass spectrometry of anti-Pol II immune-precipitates obtained from crosslinking chromatin fractions of Pol III_degron cells under untreated and 1 hr IAA treatment conditions (upper). The protein factors listed here showed a similar trend of differential interactions with Pol II in both the canonical DDA and DIA MS experiments (See the detailed procedure in the supplementary method section). The color bar indicates proteins differentially interacting with Pol II, characterized by the log2FC and -log10(P value) with DIA MS data. (bottom).

***References:***

*14.	Zhang F, Ge W, Ruan G, Cai X, Guo T: Data-Independent Acquisition Mass Spectrometry-Based Proteomics and Software Tools: A Glimpse in 2020. Proteomics 2020, 20:e1900276.*

*15.	Krasny L, Huang PH: Data-independent acquisition mass spectrometry (DIA-MS) for proteomic applications in oncology. Mol Omics 2021, 17:29-42.*

-Figure 6B: the quantitative power of western blots is very limited and the decrease in SSRP1 and SPT16 is not convincing.

***Response:*** *We agree with the reviewer that the changes in western blot data are small. Western blot experiments were performed with three independent replicates and revealed a similar decreasing trend, and two different mass spectrometry datasets also revealed a similar trend of limited decrease. As Pol III depletion affects only a small fraction of FACT chromatin binding compared to its genome-wide distribution, the changes in Pol II interaction with FACT would be diluted by the background when examined by western blot. We stated the limitation of the quantitative western blot showing a small decrease in the revised manuscript.*

On the contrary, the effect they observe on SSRP1 recruitment by ChIP is rather massive, to the point that one could ask how it is possible that this effect is linked to the decreased binding of Pol III to a relatively few sites. The conclusions would probably be more convincing if the authors showed SSRP1 ChIP-seq data for several individual examples (only Nipbl was shown and it is not clear whether Pol III binds close to its promoter). Showing the fold change (FC) or the log2 FC between treated and untreated conditions can help readers to appreciate whether the change in the signal is a real increase/decrease or just a slightly different distribution.

***Response:*** *Our original Figure 6E shows that decreased FACT binding sites were preferentially enriched in Pol III peak regions, which implies that one Pol III binding site may locally regulate FACT recruitment to multiple sites in linear or spatial proximity. This phenomenon is also reminiscent of several genes sharing the same enhancer, and multiple genomic regions may cluster in proximity to share regulators in the three-dimensional nucleus [16-18]. We also showed the specific binding of Pol III near the Nipbl and Mycbp2 gene promoters, and these signals did not appear in the input sample and could be further validated with ChIP–qPCR (Figure R6, see below). Mycbp2 also showed a slowdown of the Pol II transcription rate with the DRB inhibition/release experiment after Pol III depletion (Figure R7). Two independent replicates of the dataset consistently showed a decrease in SSRP1 binding and chromatin accessibility and increased Pol II occupancy at the gene bodies (Figure R6, R7), and read per genome coverage (RPGC)-normalized count quantification showed a significant increase in the gene body regions of Pol II ChIP-seq and PRO-seq and a decrease in the promoter regions of ATAC-seq and SSRP1 ChIP-seq upon Pol III or RPAC1 depletion at the Nipbl and Mycbp2 regions (Figure R8).*

*Furthermore, we performed SSRP1 ChIP–qPCR upon Pol III depletion as well as its recovery, which showed that SSRP1 chromatin binding decreased upon Pol III depletion and returned to normal levels when Pol III recovered (Figure R9), suggesting that Pol III regulation of FACT chromatin occupancy is specific.*

*In the revision, we replaced the track at the Nipbl region with two biological replicates and showed the Pol III binding near the promoter, as the reviewer suggested (Figure 7C), and another example at the Mycbp2 region with the transcription rate test results as supplementary data (New Figure S4C). Quantifications of RPGC normalized counts at these two regions were added as supplementary data (New Figure S4D). SSRP1 ChIP–qPCR tests at presented examples upon Pol III depletion and recovery were also added as supplementary data (New Figure 6G).*

**Figure R6**. Genome browser track at the 8,269,424-8,592,237 region on chromosome 15 for Pol II ChIP-Seq signals in Pol III_degron and RPAC1_degron cells and SSRP1 ChIP-seq signals in Pol III_degron cells under untreated and 1 hr IAA treatment conditions. PRO-seq and ATAC-seq in Pol III_degron cells under untreated and 1 hr IAA treatment conditions and Pol III ChIP-seq in wild-type mESCs are shown in the same region, and only sense strand signals of PRO-Seq and ChAR-Seq are presented and all tracks are flipped horizontally.

**Figure R7**. Upper panel: Genome browser track at the 103,104,177-103,384,370 region on chromosome 14 for Pol II ChIP-Seq signals in Pol III_degron and RPAC1_degron cells and SSRP1 ChIP-seq signals in Pol III_degron cells under untreated and 1 hr IAA treatment conditions. PRO-seq and ATAC-seq in Pol III_degron cells under untreated and 1 hr IAA treatment conditions and Pol III ChIP-seq in wild-type mESCs are shown in the same region, and only sense strand signals of PRO-Seq and ChAR-Seq are presented and all tracks are flipped horizontally. Bottom panel: Bar graphs showing relative ChIP enrichment normalized to input (5%) at the locus indicated in the upper panel. Each sample was analyzed with two technical replicates per biological replicate and two biological replicates in total. Statistical significance was evaluated by Student’s t test (**: <0.01, *: <0.05).

**Figure R8**. Reads per genome coverage (RPGC)-normalized count quantification of ATAC-seq and SSRP1 ChIP-seq at the promoter region and Pol II ChIP-seq and PRO-seq at the gene body region upon Pol III or RPAC1 depletion. Statistical significance was evaluated by Student’s t test (**: <0.01, *: <0.05).

**Figure R9**. Bar graphs showing relative SSRP1 ChIP enrichment normalized to input (5%) at the presented example genes under Pol III untreated, + IAA 1 hr and +IAA 1 hr followed by 6 hr IAA withdrawal conditions. Each sample was analyzed with two technical replicates per biological replicate and two biological replicates in total. Statistical significance was evaluated by Student’s t test (**: <0.01, *: <0.05).

***References:***

*16.	Levo M, Raimundo J, Bing XY, Sisco Z, Batut PJ, Ryabichko S, Gregor T, Levine MS: Transcriptional coupling of distant regulatory genes in living embryos. Nature 2022, 605:754-760.*

*17.	Quinodoz SA, Ollikainen N, Tabak B, Palla A, Schmidt JM, Detmar E, Lai MM, Shishkin AA, Bhat P, Takei Y, et al: Higher-Order Inter-chromosomal Hubs Shape 3D Genome Organization in the Nucleus. Cell 2018, 174:744-757.e724.*

*18.	Field A, Adelman K: Evaluating Enhancer Function and Transcription. Annu Rev Biochem 2020, 89:213-234.*

Also related to FACT recruitment, upon Pol III depletion at genebody-up genes the authors observe by ChIP decreased binding of Pol II at the promoter with concomitant decrease in SSRP1 ChIP signal and higher nucleosome occupancy. They conclude that FACT recruitment is compromised because of the absence of Pol III binding nearby and that this is the cause of the different Pol II pattern. However, it could very well be that it is the decrease in Pol II binding to the promoter or a decrease in the initiation rate that provokes a reduction in FACT recruitment and higher nucleosome persistence. Indeed, in a recent article in the yeast model, it has been proposed that FACT is recruited to the +1 nucleosome once it is partially unwrapped by Pol II (PMID: 34380014). If that were the case, their entire model would not be correct and it would remain completely unclear why Pol III depletion leads to different Pol II signals at as subset of genes.

***Response:*** *Our model is that Pol III depletion affects FACT recruitment and causes defects in nearby Pol II transcription elongation. However, another possible model is that Pol III depletion affects Pol II distribution, leading to defects in FACT recruitment, as the reviewer suggested. It is indeed quite challenging to directly test these two models. We believe that Pol III degron followed by time-series ChIP–qPCR experiments may provide useful information. We thus performed SSRP1, Pol II and Pol III ChIP–qPCR upon acute Pol III depletion at different time points (untreated, 10 min, 20 min, 30 min and 1 hr IAA treatment) to examine their chromatin binding at specific genes (Bub1, Nipbl and Mycbp2) (Figure R10). The results showed that the chromatin binding of Pol III immediately decreased after 10 min of IAA treatment, and FACT binding exhibited a significant decrease after 20 min of IAA treatment. In contrast, the Pol II distributions were significantly affected only after 30 min or 1 hr of IAA treatment. Namely, the FACT response occurred earlier than the Pol II response after Pol III depletion. In addition, we provided evidence that FACT knockdown specifically increases the transcriptional signal at the gene body of these genebody-up genes with NET-seq data, as shown in Figure 6F in the original manuscript (New Figure 6H). Together, these results were consistent with our model that Pol III depletion affects FACT recruitment, resulting in different Pol II binding patterns. However, our results could not completely rule out that the reviewer’s model also contributes to Pol III-mediated Pol II regulation.*

*There may be multiple mechanisms that could cause deficiency in nucleosome partial unwrapping, which would impair FACT recruitment in different circumstances in cells, as discussed in the reference (PMID: 34380014) that the reviewer mentioned: “Because such partial unwrapping can be achieved in multiple ways and in different circumstances in cells, the model proposed here provides an attractive recruitment mechanism for FACT to function in different contexts.” [19] It is possible that Pol III functions in the nucleosome partial unwrapping of nearby Pol II genes in specific loci. Previous studies also revealed multiple mechanisms of cross-regulation between different RNA polymerases [11-13, 20-22], such as transcriptional interference, chromatin looping* *and noncoding RNAs. We carefully subgrouped the Pol III-affected Pol II genes in Figure 4B based on different features, presented our hypothesis, and discussed that multiple models may help us to understand the cross-regulation between Pol III and Pol II. It is possible that Pol III regulates Pol II through multiple mechanisms independently and combinatorically at different genes. Thus, we have further toned down our statement to emphasize the interesting observations reported in our study, carefully concluded that our results are correlated with the FACT-Pol II axis model, and discussed that other models can also explain our results.*

*We believe that we need both our model and the reviewer’s model to fully explain the defects of Pol II after Pol III depletion. After Pol III depletion, the nucleosome partial unwrapping nearby is impaired and FACT first responds and decreases, and Pol II binding decreases in the promoter-proximal regions, as evidenced by acute degron time-series ChIP–qPCR at specific gene loci. Then, these decreased Pol II occupancies would further decrease FACT recruitment to the +1 nucleosome, as described by the reviewer. This positive feedback relationship between Pol II and FACT would cause massive defects in FACT recruitment and Pol II occupancy, as observed in our Pol II and FACT ChIP-seq datasets. We have discussed these models in our revised manuscript and appreciate the reviewer’s deep thinking on the relationship between Pol lI and the FACT complex.*

*In the revision, we added SSRP1, Pol II and Pol III ChIP–qPCR upon acute Pol III depletion at different time points as New Figure 6F.*

**Figure R10**. Bar graphs showing relative SSRP1, Pol II and Pol III ChIP enrichment normalized to input (5%) at the presented genes under Pol III untreated and + IAA 10 min, 20 min, 30 min, and 1 hr conditions. Each sample was analyzed with two technical replicates per biological replicate and two biological replicates in total. Statistical significance was evaluated by Student’s t test (***: <0.001, **: <0.01, *: <0.05).

***References:***

*11.	Abraham KJ, Khosraviani N, Chan JNY, Gorthi A, Samman A, Zhao DY, Wang M, Bokros M, Vidya E, Ostrowski LA, et al: Nucleolar RNA polymerase II drives ribosome biogenesis. Nature 2020, 585:298-302.*

*12.	Yeganeh M, Praz V, Cousin P, Hernandez N: Transcriptional interference by RNA polymerase III affects expression of the Polr3e gene. Genes Dev 2017, 31:413-421.*

*13.	Gerber A, Ito K, Chu CS, Roeder RG: Gene-Specific Control of tRNA Expression by RNA Polymerase II. Mol Cell 2020, 78:765-778.e767.*

*19.	Jeronimo C, Angel A, Nguyen VQ, Kim JM, Poitras C, Lambert E, Collin P, Mellor J, Wu C, Robert F: FACT is recruited to the +1 nucleosome of transcribed genes and spreads in a Chd1-dependent manner. Mol Cell 2021, 81:3542-3559.e3511.*

*20.	Policarpi C, Crepaldi L, Brookes E, Nitarska J, French SM, Coatti A, Riccio A: Enhancer SINEs Link Pol III to Pol II Transcription in Neurons. Cell Rep 2017, 21:2879-2894.*

*21.	Crepaldi L, Policarpi C, Coatti A, Sherlock WT, Jongbloets BC, Down TA, Riccio A: Binding of TFIIIC to sine elements controls the relocation of activity-dependent neuronal genes to transcription factories. PLoS Genet 2013, 9:e1003699.*

*22.	Zhang XO, Gingeras TR, Weng Z: Genome-wide analysis of polymerase III-transcribed Alu elements suggests cell-type-specific enhancer function. Genome Res 2019, 29:1402-1414.*

-Writing needs more than polishing. There are a number of sentences that are grammatically incorrect (e.g. lacking a verb) and paragraphs that are not clear. Some examples:

- Legend of figure 4: "the unchanged genes are genes that did not be affected by Pol III depletion at all. Detailes see Methods)"

***Response:*** *We replaced this sentence with “**the unchanged genes are defined by selecting the genes that were not at all affected by Pol III depletion. For details, see Methods.”*

- Lines 185-187: "Further overlap analyses showed that the peaks changed in the Pol II ChIP-Seq data after Pol III depletion had the most overlap with the Pol III ChIP-Seq peaks in both the ChIP-Seq and PRO-Seq datasets". I do not understand what this means.

***Response:*** *We have replaced this sentence with “**Further overlap analyses showed that the signal significantly changed after Pol III depletion in both the Pol II ChIP-seq and PRO-seq datasets and exhibited the most overlap with the Pol III ChIP-seq peaks.”*

- Lines 207-210: " The genebody-up genes were identified by overlapping genes with upregulated signals after Pol III depletion identified by both Pol II ChIP-Seq and PRO-Seq of gene bodies, and genebody-unaffected genes were identified by overlapping the genes with unchanged signals under the same conditions". This paragraph is not very clear. I suggest to reformulate as follows (if I understood well): genes with increased Pol II ChIP-seq and PRO-Seq signal at the gene body were designated genebody-up genes... etc

***Response:*** *We replaced this sentence with “**Genes with both Pol II ChIP-seq and PRO-seq signals at the gene body increased upon Pol III depletion were designated gene body-up genes, and genes with both Pol II ChIP-seq and PRO-seq signals at the gene body unchanged upon Pol III depletion were designated gene body-unaffected genes.”*

- Lines 263-265: " As retrotransposon elements SINE encoding non-coding RNAs transcribed by Pol III, and that were previously proposed to link to Pol II through chromatin looping."

***Response:*** *We replaced this sentence with “**The SINE retrotransposon elements encode Pol III-transcribed noncoding RNAs, which were proposed to regulate Pol II transcription through chromatin looping.”*

- Related to writing, most legends are not clear, so it is quite difficult to understand the data presented. Also, there are some mistakes. For instance, in the figure 4 legend they say mRNAs while they are talking about genes.

***Response:*** *We have rewritten the figure legends throughout the revised manuscript for clarity.*

Other comments/concerns:

- Line 213: Pausing index. I guess the authors mean promoter-proximal pausing index.

Related to this, their conclusion that "depletion of Pol III leads to a widespread decrease in Pol II initiation at promoter regions, while increases Pol II signals at gene bodies for a subset of genes" is weird in the light of their results. Considering that they detect either increased signal or unchanged signal at gene bodies and that the Pol II signal in gene bodies should in principle derive from initiation events, I think the only thing they can propose at this stage is that promoter-proximal pausing is decreased upon Pol III depletion.

***Response:*** *We have replaced this sentence with “**depletion of Pol III leads to a widespread decrease in promoter-proximal pausing with decreased initiation at gene promoter regions and increased Pol II signals at gene bodies for a subset of genes, as we described.”*

- Line 220: they talk about "PRO-Seq signals at the promoter" but PRO-Seq does not detect Pol II at the promoter. Since it sequences the nascent RNA, it can only detect polymerases that have initiated transcription.

***Response:*** *PRO-seq detects active Pol II (including both the initiated and elongated Pol II)-transcribed nascent RNA, as reported previously [23]. The initiated Pol II would generate nascent RNA at the promoter-proximal regions. We changed the mention of the promoter to* *promoter-proximal regions in the revised manuscript.*

***Reference:***

*23.	Kwak H, Fuda NJ, Core LJ, Lis JT: Precise maps of RNA polymerase reveal how promoters direct initiation and pausing. Science 2013, 339:950-953.*

- The authors often confound correlations with causal connections. For instance, in lines 268-271 they say "We then annotated the Pol III binding sites that have regulatory roles on Pol II, and identified 121 (15.7%), 68 (8.9%), and 34 (4.4%) genebody-up genes (N=773) that could be explained by transcriptional interference, chromatin looping, and ncRNA models". It seems to me that they just hypothesize that these genes are regulated by Pol III by the indicated mechanisms based of the position of Pol III peaks relative to each genebody-up gene. They should clarify this in the text.

***Response:*** *We appreciate and agree with the reviewer that we should only hypothesize that these genes are regulated by Pol III by the indicated mechanisms based on the position of Pol III peaks relative to each gene body-up gene. We have further toned down our statement to emphasize the interesting observations reported in our study and have carefully concluded that our results are correlated with the models rather than the casual connections.*

- Figure 6E: I'm not sure how to interpret these data, the authors could explain a bit more the rationale of this analysis and indicate more clearly what the y axis represents.

***Response:*** *We thank the reviewer for pointing out that the description of categorical distance analysis was not sufficiently clear. Several genome-wide studies have found human Pol III presence near the active Pol II gene promoters, while Pol II was also found near the majority of Pol III-occupied loci [2-4]. Intrigued by this phenomenon, we wanted to explore the relevance of Pol III peaks or tRNAs near Pol III-affected mRNA genes. Indeed, as predicted, histogram plots of Pol III peaks or tRNAs residing in different classes of Pol III-affected mRNA genes revealed that Pol II gene body-up genes usually had nearby Pol III peaks (see Figure 4A). These observations were consistent with the models that cells may use Pol III as Pol II regulators at some genes by transcriptional interference, noncoding RNAs or FACT to regulate nearby mRNA gene transcription [13, 20, 24].*

*To investigate whether the FACT and integrator complex may serve as a protein–protein interface between Pol II and Pol III concerning their occupancy at gene promoters, we performed FACT, TBP and INTS3 ChIP-Seq in IAA-treated and untreated Pol III_degron cells. Notably, the differential binding analysis revealed that at a consensus list of 2,526 Pol II-affected gene promoters, Pol III depletion caused 1,636 (64.8%) sites to have a decreased FACT signal, while only 103 (4.1%) and 63 (2.5%) sites displayed differential INT3 and TBP signals, respectively.*

*Given the substantial overlap of Pol II and FACT interference with Pol III, we further tested the association between the Pol III peaks or tRNAs and the presence of FACT-, TBP- or INTS3-affected sites in the respective distance bin with the same approach as above (Figure 4A). By comparing the occurrence of Pol III-bound peaks and expressed tRNAs in the three gene categories, we observed a clear preference for Pol III peak enrichment at FACT-affected sites but not at the TBP- and INTS3-affected sites, as shown in Figure 6E. We regret confusing the reviewer and have made this point clear in the revised manuscript.*

*The y-axis in Figure 6E represents the normalized counts of Pol III peaks or tRNAs by calculating their average density within 10 kb of the Pol II gene TSS for each category. These histogram plots can be considered ChIP-seq average peak-density profiles. We have added text to clarify this point and modified the legend to note the Y-axis for the histograms in Figure 6E.*

***References:***

*2.	Barski A, Chepelev I, Liko D, Cuddapah S, Fleming AB, Birch J, Cui K, White RJ, Zhao K: Pol II and its associated epigenetic marks are present at Pol III-transcribed noncoding RNA genes. Nat Struct Mol Biol 2010, 17:629-634.*

*3.	Moqtaderi Z, Wang J, Raha D, White RJ, Snyder M, Weng Z, Struhl K: Genomic binding profiles of functionally distinct RNA polymerase III transcription complexes in human cells. Nat Struct Mol Biol 2010, 17:635-640.*

*4.	Oler AJ, Alla RK, Roberts DN, Wong A, Hollenhorst PC, Chandler KJ, Cassiday PA, Nelson CA, Hagedorn CH, Graves BJ, Cairns BR: Human RNA polymerase III transcriptomes and relationships to Pol II promoter chromatin and enhancer-binding factors. Nat Struct Mol Biol 2010, 17:620-628.*

*13.	Gerber A, Ito K, Chu CS, Roeder RG: Gene-Specific Control of tRNA Expression by RNA Polymerase II. Mol Cell 2020, 78:765-778.e767.*

*20.	Policarpi C, Crepaldi L, Brookes E, Nitarska J, French SM, Coatti A, Riccio A: Enhancer SINEs Link Pol III to Pol II Transcription in Neurons. Cell Rep 2017, 21:2879-2894.*

*24.	Tettey TT, Gao X, Shao W, Li H, Story BA, Chitsazan AD, Glaser RL, Goode ZH, Seidel CW, Conaway RC, et al: A Role for FACT in RNA Polymerase II Promoter-Proximal Pausing. Cell Rep 2019, 27:3770-3779.e3777.*

**Reviewer #2:** In this paper the authors use the degron system to tag and rapidly degrade the primary subunit of RNA polymerase I, II, and III. The authors show clear evidence that their degron tag works to degrade the appropriate RNA polymerase submit. They use these cell lines, in conjunction with ChIP-seq, PRO-seq, and Hi-Chip data to explore how perturbation of RNA polymerase I, II, and III affects each of the other RNA polymerases. The authors generally find that Pol III has an impact on Pol II transcription in a variety of locations across the genome, including some previously identified loci. The authors go on to argue that Pol III depletion affects elongation rate through FACT deposition in Pol II. This paper is interesting, and in general it is well-written. Results are generally well-supported by the data. My main comment is that the writing is dense, and some of the results are difficult to follow. I would encourage the authors to go through the manuscript with an eye toward additional clarity of the presentation.

***Response:*** *We appreciate the reviewer’s comment that our paper is interesting. We added more explanation and discussion to clarify our results and conclusions, as the reviewer suggested.*

Additional comments:

Could ChIP-seq sites in which all three RNA Pols overlap be driven by mapping artifacts? Have ENCODE blacklist regions (and other similar sites) been removed prior to analysis?

***Response:*** *We thank the reviewer for raising this important issue. Mapping artifacts mainly arise from PCR amplification bias and aberrant high signals within problematic regions [25, 26].* *To rule out the possibility that mapping artifacts might drive the overlapping ChIP-seq peaks of all three RNA Pols, we added details of our ChIP-seq processing steps described earlier [1] and performed an additional quality examination of aligned reads. Following the guidelines and quality control (QC) measures of the ENCODE consortia, we implemented the ChIP-seq analysis pipeline consisting of four main QC steps as follows:*

1. *The adaptor and low-quality reads were filtered out using cutadapt (v2.10).*
2. *Unmapped reads and multiple mapped reads were removed using SAMtools (v 0.1.19).*
3. *PCR duplicates were removed using sambamba (v0.7.0) [27, 28]; only uniquely mapped reads were retained before peak calling with MACS2.*
4. *Peaks that overlapped with the ENCODE blacklisted regions (https://github.com/Boyle-Lab/Blacklist/) [29] were excluded before further analysis.*

*In addition, the quality of each RNA Pol library was assessed in three ways. First, we quantified the fragment length signal and the ratio of fragment length signal to read length signal with run_spp. R from the phantompeakqualtools program (v1.15) and observed that all libraries passed the ENCODE quality criteria (NSC > = 1.05; RSC > = 0.9) (Figure R11, B) [30]. Second, we generated a report containing ChIP-seq quality metrics and diagnostic graphics using ChIPQC (v1.33) with default options [31], and all libraries obtained good QC scores (relatively high RiP and SSD values, see below table). Third, we manually inspected the read coverage plots and peak distributions of the three RNA Pols to ensure that there were no technical artifacts (such as coverage inequality and uneven fragmentation efficiency) that incur problems with peak calling (Figure R11, C and D).*

*With the new QC analysis, we have provided a more detailed description of the processing procedure in the Methods section and quality control metrics in the Supplementary Table. We hope our response and revised manuscript will resolve the reviewer’s concerns.*

*In the revision, we added the quality control results as New Figure S1B, C, D.*

**Figure R11**. Quality control of ChIP-seq datasets and Pol I, II, and III ChIP-Seq was performed in mES cells in two replicates. B: An individual cross-correlation plot for the respective ChIP-Seq sample is shown with NSC and RSC values indicated below, suggesting that our data are of high quality. NSC represents the normalized strand coefficient, while RSC represents the relative strand coefficient. C: The coverage histogram depicting the sequencing depths for each RNA Pol of two independent replicates. D: Heatmap showing the relative enrichment of genomic features. Bottom: Detailed quality control metrics. The table shows ChIP-seq quality metrics and diagnostic graphics using ChIPQC (v1.33) with the default option.

***References:***

*25.	Park PJ: ChIP-seq: advantages and challenges of a maturing technology. Nat Rev Genet 2009, 10:669-680.*

*26.	Bailey T, Krajewski P, Ladunga I, Lefebvre C, Li Q, Liu T, Madrigal P, Taslim C, Zhang J: Practical guidelines for the comprehensive analysis of ChIP-seq data. PLoS Comput Biol 2013, 9:e1003326.*

*27.	Tarasov A, Vilella AJ, Cuppen E, Nijman IJ, Prins P: Sambamba: fast processing of NGS alignment formats. Bioinformatics 2015, 31:2032-2034.*

*28.	Li H, Handsaker B, Wysoker A, Fennell T, Ruan J, Homer N, Marth G, Abecasis G, Durbin R, Genome Project Data Processing S: The Sequence Alignment/Map format and SAMtools. Bioinformatics 2009, 25:2078-2079.*

*29.	Amemiya HM, Kundaje A, Boyle AP: The ENCODE Blacklist: Identification of Problematic Regions of the Genome. Sci Rep 2019, 9:9354.*

*30.	Landt SG, Marinov GK, Kundaje A, Kheradpour P, Pauli F, Batzoglou S, Bernstein BE, Bickel P, Brown JB, Cayting P, et al: ChIP-seq guidelines and practices of the ENCODE and modENCODE consortia. Genome Res 2012, 22:1813-1831.*

*31.	Carroll TS, Liang Z, Salama R, Stark R, de Santiago I: Impact of artifact removal on ChIP quality metrics in ChIP-seq and ChIP-exo data. Front Genet 2014, 5:75.*

Line 287 - the topic starts with decreased nucleosome positioning, but no data is shown (only general accessibility). Are the authors talking about accessibility (i.e., the amount of signal)? Or nucleosome positioning?

***Response:*** *We thank the reviewer for the reminder. TSS chromatin accessibility and nucleosome positioning were both assessed from ATAC-seq data. Because ATAC-seq does not apply rigorous size selection during library preparation, people have observed that in a standard ATAC-seq library, short fragments are concentrated in nucleosome-free regions (NFRs, which represent accessible chromatin), whereas long fragments are enriched in nucleosome-associated DNA [32, 33], which represent nucleosome positioning information. Therefore, the fragment-size distribution can be modeled as a mixture of nucleosome-free and nucleosome-associated fragments in a way that captures the highly structured nature of the nucleosomal distribution, and many analysis tools have been developed to detect regions where these long nucleosomal fragments are enriched, such as DANPOS2[34], NucTools [35], and nucleoATAC [36].*

*To distinguish between chromatin accessibility differences and nucleosome positioning changes around promoter regions, ATAC-seq data were pooled between replicates, and we used nucleoATAC (v0.3.4) with default parameters to output nucleosome signal tracks and call nucleosome dyads and nucleosome positioning. After ATAC-seq data processing and alignment, RPGC-normalized signal tracks representing chromatin accessibility were generated from replicate-merged data using deepTools bamCoverage with the parameters "–normalizeUsingRPGC –bs 10". For details, see the Methods section. The results show that Pol III depletion specifically increases nucleosome positioning at gene body-up genes, while Pol II depletion specifically increases nucleosome positioning at downregulated tRNAs. Despite lower resolution than MNase-seq, ATAC-seq could also offer more chromatin accessibility information, as the reviewer reminded us. We have described this limitation as advised by the reviewer.*

***References:***

*32.	Buenrostro JD, Giresi PG, Zaba LC, Chang HY, Greenleaf WJ: Transposition of native chromatin for fast and sensitive epigenomic profiling of open chromatin, DNA-binding proteins and nucleosome position. Nat Methods 2013, 10:1213-1218.*

*33.	Yan F, Powell DR, Curtis DJ, Wong NC: From reads to insight: a hitchhiker's guide to ATAC-seq data analysis. Genome Biol 2020, 21:22.*

*34	Chen K, Xi Y, Pan X, Li Z, Kaestner K, Tyler J, Dent S, He X, Li W: DANPOS: dynamic analysis of nucleosome position and occupancy by sequencing. Genome Res 2013, 23:341-351.*

*35.	Vainshtein Y, Rippe K, Teif VB: NucTools: analysis of chromatin feature occupancy profiles from high-throughput sequencing data. BMC Genomics 2017, 18:158.*

*36.	Schep AN, Buenrostro JD, Denny SK, Schwartz K, Sherlock G, Greenleaf WJ: Structured nucleosome fingerprints enable high-resolution mapping of chromatin architecture within regulatory regions. Genome Res 2015, 25:1757-1770.*

Pp. 6: The switch in language from "clusters" to "groups" is confusing. Consider renaming "group x" to "cluster x".

***Response:*** *We replaced " groups" with " clusters " in the revised manuscript.*

**Second round of review**

**Reviewer 1**

The authors have made a remarkable effort in the revision of their manuscript and have certainly succeeded in addressing some important concerns I had. However, I still find a few points that in my opinion should be resolved before publication.

Major points:
-The decrease in the PRO-Seq and ChAR-seq signal at Pol III peaks upon Pol III depletion (fig S2D) is very moderate compared to the decrease observed at a model tRNA in figure 1H, so I do not think these data validate the idea that the peaks observed are bona fide new Pol III transcription units. Maybe showing some genome browser snapshots would be more convincing.

-Figure 5 and S3C: if Pol II depletion provokes downregulation of an activator of Pol III genes (C-MYC), it is very difficult to conclude that Pol II-mediated nucleosome architecture partially contributes to the transcription of specific tRNAs. Are all tRNAs affected by Pol II depletion dependent on C-MYC? Is it really significant the decrease in C-MYC expression during the timeframe of the experiment? If the response to both questions is yes, the authors would need to perform additional experiments in the presence of additional C-MYC copies or C-MYC expressed from a system that bypasses repression upon Pol II depletion to evaluate what part of the effect observed is indeed potentially due to changes in nucleosome architecture.

-Figure 6B: The authors have now indicated that the western blot analyses presented were performed 3 times. However, they do not mention whether the values indicated correspond to the average of the three replicates, what is the standard deviation and whether they applied a statistical test that indicated significant differences between the samples. If, as the authors suggest, Pol III depletion lead to decreased association of only a fraction of the Pol II pool with FACT, it would be conceivable that such differences could not be detected by IP followed by western blot. If indeed the IP-western blot analyses are not conclusive these data should be removed from the main figure and in the main text they should rather say something like "No robust decrease in the association of Pol II with FACT could be detect by IP followed by western blot, possible because Pol III depletion affects the interaction of only a relatively small fraction of cellular Pol II".

-Lines 1273- 1278: I do not get this reasoning. To me if transcription by Pol II is affected by depletion of Pol III, transcription-dependent chromatin marks should anyway be altered. It seems to me that the experiment mentioned in the above section (Pol III, Pol II and FACT ChIP analyses during time-course depletion of Pol III, figure 6H) is a better support for the idea that changes in the chromatin structure precede -and, thus, are likely the cause of- Pol II transcriptional changes than the experiment in figure 7A (i.e. absence of changes in H3K36me3 levels).

-Figure 7E: the authors now show RNAseq experiments conducted after 24h, 48h and 96h depletion of Pol III. Such prolonged depletion of Pol III is expected to provoke a general downregulation of gene expression associated with the reduction/arrest of cell proliferation. Indeed, repression of not only genebody-up genes but also many others was most likely observed in these experiments (although they only showed the results for the genebody-up genes). Actually, the authors conducted their ChIP-Seq and PRO-Seq experiments upon acute Pol III depletion to avoid this kind of confounding effects. Therefore, the new data do not particularly support the idea that Pol III binding to chromatin effectively regulates the expression of specific Pol II genes (i.e. genebody-up genes). I understand that proving this regulation is complicated because the half-life of mouse mRNAs is relatively long (12-15h), and, thus, moderate changes in the production of RNA would not be detectable by RNA-seq within the time frame of acute Pol III depletion (i.e. 1h). A way to resolve this could be to use their analyses of newly synthetized RNAs (EU-Seq). In figure 7B they show a decrease in the EU-Seq/Pol II ChIP-Seq signal ratio, but do they observe also a net decrease of the EU-Seq signal? (they should if RNA production is indeed compromised).

-In all genome browser tracks, the contrast between the colours used (blue and the dark red) is very low, making visualization of overlapped conditions (+/- IAA) difficult. Maybe using lines instead of histograms would help.

-Regarding manuscript writing, although many parts have improved, I recommend one extra round of corrections since many errors persist and sometimes this complicates comprehension of the text. A non-exhaustive list of examples is here below:

-In the abstract background I suggest the following rephrasing: " Mammalian nuclei include RNA polymerases (Pols) I, II, and III. The extent to which these polymerases are cross-regulated and the involved mechanisms remain unclear".

-Line 865: …Pol II transcribes transfer RNA (tRNA) and small RNA… should be changed to "Pol III synthetises transfer RNAs (tRNAs) and other small non-coding RNAs"

-Line 900: an extra "Pols" in the sentence to remove.

-Line 906: "Pol II ChIP following mass spectroscopy" should be changed to "Pol II ChIP followed by mass spectroscopy.

-Line 990: Substitute "Although" by "However".

-Line 999: Substitute "cross-regulatory" by "cross-regulation".

-Lines 1026-1029: the new sentence is not very clear.

-Lines 1064-1066: It is unclear to me what this sentence means.

-Lines 1121-1122: there is an extra "nearby" to remove from this sentence.

-Line 1172: "had" should be removed from this sentence.

-Lines 1197-1198: One of the two "independently" should be deleted.

-Lines 1271-1273: this sentence is not clear (likely grammatically incorrect).

-Lines 1373-1391: the new inserted text has multiple grammatical errors.

A few additional minor points:

-Line 888-889: because RNA pol III activity is essential for cell proliferation and survival, I guess the authors meant that "a reduction" or "partial inhibition" (and not inhibition) of Pol III activity extends yeast and drosophila lifespan.

-Lines 1211-1213: to my knowledge Integrator is not known to regulate Pol III transcription.

-Line 947-948: ChIP-seq provides a readout of proteins binding to chromatin, not to RNA. Therefore I suggest the following correction: "As expected, Pol I, Pol II and Pol III ChIP-seq signals where enriched differentially at loci producing known RNAs such as rRNAs…"

-Lines 1061-1063: I still think that the Pol II ChIP-seq pattern observed upon depletion of Pol III suggests decreased promoter-proximal pausing but does not necessarily indicate decreased initiation.

-Figure 5A: Neither in the text nor in the legend of figure 5 it is indicated whether the PRO-Seq experiment performed upon Pol II depletion is on Pol III (I assumed it is the case).

**Reviewer 3**

The authors have carefully addressed the reviewers' comments.

**Authors Response**

**Point-by-point responses to the reviewers’ comments:**

Reviewer reports:

Reviewer #1: The authors have made a remarkable effort in the revision of their manuscript and have certainly succeeded in addressing some important concerns I had. However, I still find a few points that in my opinion should be resolved before publication.

Major points:

-The decrease in the PRO-Seq and ChAR-seq signal at Pol III peaks upon Pol III depletion (fig S2D) is very moderate compared to the decrease observed at a model tRNA in figure 1H, so I do not think these data validate the idea that the peaks observed are bona fide new Pol III transcription units. Maybe showing some genome browser snapshots would be more convincing.

***Response:*** *We agree with the reviewer that the changes in ChAR-seq are indeed moderate, as we realized that chromatin-associated RNAs consist of nascent RNAs but also contain mature RNAs associated with chromatin. This could be why we did not observe a more dramatic decrease in the ChAR-seq signals. For this reason, we replaced the ChAR-seq with the EU-seq analyses, as EU-seq assesses the nascent RNAs synthesized in cells, and the signal decreased more than that of the ChAR-seq. On the other hand, the auxin-induced degradation (AID) technique can acutely degrade the targeted protein but does not eliminate the protein. The degradation efficiency depends on the abundance and stability of the targeted proteins and the expression level of the plant derived E3 ubiquitin ligase TIR1 in the cell line. Our PRO-seq and EU-seq experiments were carried out after 1 hr of IAA treatment, which may not be long enough to have a more potent decrease. Nevertheless, this AID technique would reveal immediate effects essential for clarifying RNA polymerases' immediate functions. Furthermore, we show several browser snapshots as the reviewer suggested, and all of them show signal decreases as the metaplot indicated (Figure R1, bottom panel).*

*In the revision, we replaced the heatmap of the ChAR-seq signal with that of EU-seq at identified Pol III peaks upon Pol III depletion (New Figure S2D, upper panel), which showed a similar significant decrease upon Pol III depletion as PRO-seq. In addition, we verified Pol III binding with several browser snapshots, which showed efficient Pol III transcriptional repression upon 1 hr of IAA treatment (New Figure S2D, bottom panel).*

**Figure R1.** Upper panel, heatmap of the normalized Pol III degron PRO-seq and EU-seq signals centered at the Pol III peaks (n = 1845), showing the marked reduction in Pol III binding peaks upon its depletion. Heatmaps are ordered by descending Pol III ChIP-seq signal intensity; bottom panel, genome browser track of PRO-seq and EU-seq under untreated and after 1 hr of IAA treatment conditions in several identified Pol III peaks in Pol III degron cells. The y-axis shows the normalized read density in reads per genome coverage (RPGC).

-Figure 5 and S3C: if Pol II depletion provokes downregulation of an activator of Pol III genes (C-MYC), it is very difficult to conclude that Pol II-mediated nucleosome architecture partially contributes to the transcription of specific tRNAs. Are all tRNAs affected by Pol II depletion dependent on C-MYC? Is it really significant the decrease in C-MYC expression during the timeframe of the experiment? If the response to both questions is yes, the authors would need to perform additional experiments in the presence of additional C-MYC copies or C-MYC expressed from a system that bypasses repression upon Pol II depletion to evaluate what part of the effect observed is indeed potentially due to changes in nucleosome architecture.

***Response:*** *The quantification of C-MYC levels with the Western blot showed that C-MYC proteins decreased ~20% upon Pol II depletion for 1 hr. We then downloaded three independent C-MYC ChIP-seq datasets in mESCs from the ENCODE database (GSM288356, GSM3103385, GSM2417145) and found that C-MYC peaks did not overlap with our down- and upregulated tRNAs (Figure R2). These results indicated that the slight decrease of C-MYC may not simply explain the effects of tRNA expression after the loss of Pol II. On the other hand, it is also possible that the chromatin binding of C-MYC at these tRNAs is weak or that these tRNAs might be less sensitive to the slight reduction in C-MYC due to the acute degradation of Pol II for 1 hr. Thus, we stated that the Pol II-mediated local nucleosome architecture may partially contribute to the transcription of specific tRNAs. However, we cannot completely rule out the possibility that C-MYC may directly or indirectly affect tRNA expression in the revised manuscript.*

*In the revision, we added colocalization analysis between C-MYC peaks and tRNAs with differential expression upon Pol II depletion (New figure S3D) and showed no overlap.*

**Figure R2.** Four-way Venn diagram comparing the overlap of three independent C-MYC binding peaks from publicly available ChIP-seq data in mESCs and down- (left) and upregulated (right) tRNAs from Pol II degron PRO-seq.

-Figure 6B: The authors have now indicated that the western blot analyses presented were performed 3 times. However, they do not mention whether the values indicated correspond to the average of the three replicates, what is the standard deviation and whether they applied a statistical test that indicated significant differences between the samples. If, as the authors suggest, Pol III depletion lead to decreased association of only a fraction of the Pol II pool with FACT, it would be conceivable that such differences could not be detected by IP followed by western blot. If indeed the IP-western blot analyses are not conclusive these data should be removed from the main figure and in the main text they should rather say something like "No robust decrease in the association of Pol II with FACT could be detect by IP followed by western blot, possible because Pol III depletion affects the interaction of only a relatively small fraction of cellular Pol II".

***Response:*** *The quantification value of the Western blot is not based on average but on the representative figure shown, and we have clarified this in the revised manuscript. We performed three independent Western blotting analyses. Each showed a similar trend regarding a decreased interaction between FACT and Pol II upon Pol III depletion, which is consistent with our ChIP-MS and ChIP-seq results (Figure R3). In addition, SSRP1 showed a significantly decreased interaction with Pol II upon Pol III depletion (T test, P=0.008884). Thus, we performed SSRP1 ChIP-seq after Pol III depletion. We also agree with the reviewer that the technical limitations of Western blot, such as low detection of molecules with high molecular weights or poor antibody sensitivity, may cause experimental variations. We have made this clear in the revised manuscript.*

**Figure R3.** Western blotting analysis of SSRP1, SPT16, INTS3, and INTS11 with Pol II immunoprecipitates obtained from crosslinking chromatin fractions of Pol III_degron cells under untreated and 1 hr IAA treatment conditions. The quantitative data shown under each panel were measured by ImageJ and normalized to the input under untreated conditions, respectively (figure only for the reviewer).

-Lines 1273- 1278: I do not get this reasoning. To me if transcription by Pol II is affected by depletion of Pol III, transcription-dependent chromatin marks should anyway be altered. It seems to me that the experiment mentioned in the above section (Pol III, Pol II and FACT ChIP analyses during time-course depletion of Pol III, figure 6H) is a better support for the idea that changes in the chromatin structure precede -and, thus, are likely the cause of- Pol II transcriptional changes than the experiment in figure 7A (i.e. absence of changes in H3K36me3 levels).

***Response:*** *We agree with the reviewer that Pol III, Pol II, and FACT ChIP analyses during time-course depletion of Pol III (Figure 6F) could better support our discussion here, and we corrected that in the revised manuscript. In addition, we carefully checked published literature and did not find that transient inhibition of transcription affected histone modification based on the references* *we checked. We believe that histone modification would gradually change upon Pol II depletion over time, and our short-term efficient transcriptional repression strategy could provide new clues for investigating this relationship. We have carefully discussed this in the revised manuscript.*

-Figure 7E: the authors now show RNAseq experiments conducted after 24h, 48h and 96h depletion of Pol III. Such prolonged depletion of Pol III is expected to provoke a general downregulation of gene expression associated with the reduction/arrest of cell proliferation. Indeed, repression of not only genebody-up genes but also many others was most likely observed in these experiments (although they only showed the results for the genebody-up genes). Actually, the authors conducted their ChIP-Seq and PRO-Seq experiments upon acute Pol III depletion to avoid this kind of confounding effects. Therefore, the new data do not particularly support the idea that Pol III binding to chromatin effectively regulates the expression of specific Pol II genes (i.e. genebody-up genes). I understand that proving this regulation is complicated because the half-life of mouse mRNAs is relatively long (12-15h), and, thus, moderate changes in the production of RNA would not be detectable by RNA-seq within the time frame of acute Pol III depletion (i.e. 1h). A way to resolve this could be to use their analyses of newly synthetized RNAs (EU-Seq). In figure 7B they show a decrease in the EU-Seq/Pol II ChIP-Seq signal ratio, but do they observe also a net decrease of the EU-Seq signal? (they should if RNA production is indeed compromised).

***Response:*** *We agree with the reviewer that long-term RNA-seq would lead to many indirect effects, referring to the impact of Pol III on Pol II transcription. As the reviewer suggested, we used EU-seq, which captures nascent RNA in cells and could provide more direct evidence. As stated in the last version, Pol III depletion slows down the Pol II transcription rate, decreasing the number of its transcripts. We plotted the EU-seq signal at the genebody-up and genebody-unaffected gene sets. Both the Pol III degron and its small subunit RPAC1 degron significantly decreased EU-seq signals at these genebody -up gene sets. It is worth mentioning that PRO-seq seemed to show the opposite pattern at these sites. In PRO-seq, nuclei from cells are rapidly isolated and permeabilized to wash away endogenous nucleotides as well as free RNA polymerases to halt transcription. Only RNA polymerases that remain engaged in the DNA retain their enzymatic activity and could undergo a subsequent 3-minute run-on assay. The reduced Pol II transcription rate caused Pol II to accumulate in the gene body, accumulate its transcripts and increase PRO-seq signals. However, EU-seq is performed with living cells that could complete multiple rounds of transcription during our 1 hr experimental time frame, and slowing down the transcription rate would decrease nascent RNA production. The results of both PRO-seq and EU-seq analyses are consistent with the idea that the Pol II transcription rate slows down for the genebody-up gene set upon Pol III depletion, interfering with Pol II function as a result.*

*In the revision, we added the average metagene profiles of EU-seq at genebody-up and genebody-unaffected mRNAs upon depletion of Pol III and its small subunit RPAC1 (New Figure S4E), both of which showed decreased nascent RNA transcription.*

**Figure R4.** Metagene profiles of EU-Seq signals over the genebody-up (blue) and genebody-unaffected (green) genes in IAA-treated versus untreated RPC1_degron (upper panel) and RPAC1_degron cells (lower panel), with violin plots shown on the right measuring the changes in ChIP-seq signals between IAA-treated (blue) and untreated (red) samples for genebody-up and -unaffected gene sets. P values were calculated using a Wilcoxon rank-sum test. The y-axis displays the normalized read density in reads per genome coverage (RPGC).

-In all genome browser tracks, the contrast between the colours used (blue and the dark red) is very low, making visualization of overlapped conditions (+/- IAA) difficult. Maybe using lines instead of histograms would help.

***Response:*** *In all genome browser tracks, we set tracks of +IAA condition as red color with 50% transparency, overlap with untreated (blue) condition is shown in dark red, while the +IAA section is light red. We have also tried line graphs, but histograms turned out to be the optimal choice.*

-Regarding manuscript writing, although many parts have improved, I recommend one extra round of corrections since many errors persist and sometimes this complicates comprehension of the text. A non-exhaustive list of examples is here below:

-In the abstract background I suggest the following rephrasing: " Mammalian nuclei include RNA polymerases (Pols) I, II, and III. The extent to which these polymerases are cross-regulated and the involved mechanisms remain unclear".

***Response:*** *We thank the reviewer for the suggestion, and we have replaced the text.*

-Line 865: …Pol II transcribes transfer RNA (tRNA) and small RNA… should be changed to "Pol III synthetises transfer RNAs (tRNAs) and other small non-coding RNAs"

***Response:*** *We thank the reviewer for the suggestion, and we have corrected the text.*

-Line 900: an extra "Pols" in the sentence to remove.

***Response:*** *We thank the reviewer for the suggestion, and we have deleted the text.*

-Line 906: "Pol II ChIP following mass spectroscopy" should be changed to "Pol II ChIP followed by mass spectroscopy.

***Response:*** *We thank the reviewer for the suggestion, and we have corrected the text.*

-Line 990: Substitute "Although" by "However".

***Response:*** *We thank the reviewer for the suggestion, and we have replaced the text.*

-Line 999: Substitute "cross-regulatory" by "cross-regulation".

***Response:*** *We thank the reviewer for the suggestion, and we have replaced the text.*

-Lines 1026-1029: the new sentence is not very clear.

***Response:*** *We replaced this sentence with “PRO-seq and Pol II ChIP-seq after Pol III depletion showed that the significantly altered sites overlapped more with Pol III ChIP-seq peaks than with other Pols peaks.” in the revised manuscript.*

-Lines 1064-1066: It is unclear to me what this sentence means.

***Response:*** *We clarified this by adding this sentence at the end: “PRO-seq signals at the promoter-proximal regions increased, which may be due to insufficient fragmentation during library preparation of PRO-seq causing promoter-proximal region-derived RNA to be captured with gene body-derived RNA.”*

-Lines 1121-1122: there is an extra "nearby" to remove from this sentence.

***Response:*** *We thank the reviewer for the correction, and we have deleted the text.*

-Line 1172: "had" should be removed from this sentence.

***Response:*** *We thank the reviewer for the correction, and we have deleted the text.*

-Lines 1197-1198: One of the two "independently" should be deleted.

***Response:*** *We thank the reviewer for the correction, and we have deleted the text.*

-Lines 1271-1273: this sentence is not clear (likely grammatically incorrect).

***Response:*** *We replaced this sentence with “It is quite challenging to determine whether Pol III’s effects on the chromatin structure of nearby Pol II genes are a cause or consequence of Pol II* *transcription inhibition.” in the revised manuscript.*

-Lines 1373-1391: the new inserted text has multiple grammatical errors.

***Response:*** *We have revised this part carefully.*

A few additional minor points:

-Line 888-889: because RNA pol III activity is essential for cell proliferation and survival, I guess the authors meant that "a reduction" or "partial inhibition" (and not inhibition) of Pol III activity extends yeast and drosophila lifespan.

***Response:*** *We thank the reviewer for the suggestions, and we used “a reduction” in the revised manuscript.*

-Lines 1211-1213: to my knowledge Integrator is not known to regulate Pol III transcription.

***Response:*** *We have deleted the statement related to integrator in the revised manuscript.*

-Line 947-948: ChIP-seq provides a readout of proteins binding to chromatin, not to RNA. Therefore I suggest the following correction: "As expected, Pol I, Pol II and Pol III ChIP-seq signals where enriched differentially at loci producing known RNAs such as rRNAs…"

***Response:*** *We corrected this part as the reviewer suggested.*

-Lines 1061-1063: I still think that the Pol II ChIP-seq pattern observed upon depletion of Pol III suggests decreased promoter-proximal pausing but does not necessarily indicate decreased initiation.

***Response:*** *We corrected this part as the reviewer suggested.*

-Figure 5A: Neither in the text nor in the legend of figure 5 it is indicated whether the PRO-Seq experiment performed upon Pol II depletion is on Pol III (I assumed it is the case).

***Response:*** *PRO-Seq can detect tRNAs. Figure 5A is the scatter plot displaying the tRNA expression changes with Pol I, Pol II, or Pol III depletion PRO-Seq data. We have clarified this in the revised figure legend.*

**Reviewer #3:** The authors have carefully addressed the reviewers' comments.

***Response:*** *Thank you for reviewing our manuscript.*

**Third round of review**

**Reviewer 1**

In this new revised version the authors have satisfactorily addressed my concerns.
